# Proteostasis and lysosomal repair deficits in transdifferentiated neurons of Alzheimer’s disease

**DOI:** 10.1038/s41556-025-01623-y

**Published:** 2025-03-26

**Authors:** Ching-Chieh Chou, Ryan Vest, Miguel A. Prado, Joshua Wilson-Grady, Joao A. Paulo, Yohei Shibuya, Patricia Moran-Losada, Ting-Ting Lee, Jian Luo, Steven P. Gygi, Jeffery W. Kelly, Daniel Finley, Marius Wernig, Tony Wyss-Coray, Judith Frydman

**Affiliations:** 1 Department of Biology, Stanford University, Stanford, CA USA; 2 Aligning Science Across Parkinson’s (ASAP) Collaborative Research Network, Chevy Chase, MD USA; 3 Department of Chemical Engineering, Stanford University, Stanford, CA USA; 4 Department of Neurology and Neurological Sciences and The Phil and Penny Knight Initiative for Brain Resilience, Stanford University, Stanford, CA USA; 5 Qinotto Inc., San Carlos, CA USA; 6 Department of Cell Biology, Harvard Medical School, Boston, MA USA; 7 Instituto de Investigación Sanitaria del Principado de Asturias (ISPA), Oviedo, Spain; 8 Department of Pathology, Stanford University School of Medicine, Stanford, CA USA; 9 Institute for Stem Cell Biology and Regenerative Medicine, Stanford University School of Medicine, Stanford, CA USA; 10 Wu Tsai Neurosciences Institute, Stanford University School of Medicine, Stanford, CA USA; 11 Palo Alto Veterans Institute for Research Inc. (PAVIR), Palo Alto, CA USA; 12 Department of Chemistry, The Scripps Research Institute, La Jolla, CA USA; 13 The Skaggs Institute for Chemical Biology, The Scripps Research Institute, La Jolla, CA USA; 14 Department of Genetics, Stanford University, Stanford, CA USA

**Keywords:** Organelles, Protein folding

## Abstract

Ageing is the most prominent risk factor for Alzheimer’s disease (AD). However, the cellular mechanisms linking neuronal proteostasis decline to the characteristic aberrant protein deposits in the brains of patients with AD remain elusive. Here we develop transdifferentiated neurons (tNeurons) from human dermal fibroblasts as a neuronal model that retains ageing hallmarks and exhibits AD-linked vulnerabilities. Remarkably, AD tNeurons accumulate proteotoxic deposits, including phospho-tau and amyloid β, resembling those in APP mouse brains and the brains of patients with AD. Quantitative tNeuron proteomics identify ageing- and AD-linked deficits in proteostasis and organelle homeostasis, most notably in endosome–lysosomal components. Lysosomal deficits in aged tNeurons, including constitutive lysosomal damage and ESCRT-mediated lysosomal repair defects, are exacerbated in AD tNeurons and linked to inflammatory cytokine secretion and cell death. Providing support for the centrality of lysosomal deficits in AD, compounds ameliorating lysosomal function reduce amyloid β deposits and cytokine secretion. Thus, the tNeuron model system reveals impaired lysosomal homeostasis as an early event of ageing and AD.

## Abstract (web summary)

Chou et al. generate transdifferentiated human neurons from fibroblasts from young or aged individuals and patients with Alzheimer’s disease. Studies of endolysosomes and proteostasis suggest a role for lysosomal quality control in Alzheimer’s disease pathology.

## Main

Ageing is central to Alzheimer’s disease (AD) and linked to a decline in protein homeostasis (proteostasis) and organelle homeostasis^1,2^, including the endosome–lysosome^3–6^. The mechanistic underpinnings of these defects during human brain ageing and disease remain poorly understood. Genetic models for AD that accumulate amyloid β (Aβ) or tau aggregates show endosome–lysosomal dysfunction^2,7–9^. It is a long-standing hypothesis that AD pathologies are mediated by non-cell-autonomous effects whereby extracellular aggregates taken up by neurons lead to lysosomal damage and cell death^10–13^. However, this hypothesis does not explain early disease events leading to initial dysfunction, highlighting the unmet need for patient neuronal models to better understand the molecular origins of ageing and AD pathological processes.

Developing cellular systems to study proteostasis and organellar phenotypes caused by ageing and AD in human neurons remains challenging. Although human neurons from post-mortem brains are widely studied in single-cell transcriptomics^14^, changes in the cytonuclear and organellar proteostasis networks are often not apparent from these datasets. Another common tool is induced pluripotent stem cells and their derived lineages^15^. However, this process restores youthfulness to the induced neurons, forgoing the key contribution of ageing to neurodegeneration^15,16^. The recent development of transdifferentiated neurons (herein tNeurons) directly from human adult somatic cells enables the study of neurodegenerative diseases while maintaining the contribution of ageing^16,17^. The tNeurons retain ageing and disease phenotypes^18–21^ but, due to limiting samples, they are primarily examined via transcriptomics, which do not generally reflect proteome- and organelle-wide changes. We improved the transdifferentiation approach to generate tNeurons from human dermal fibroblasts and performed quantitative proteomic analyses combined with biochemical and functional analyses comparing tNeurons obtained from healthy young and aged donors as well as from patients with sporadic AD (sAD) and familial AD (fAD). We find that ageing and AD create a cell-autonomous vulnerable state in neurons characterized by constitutive lysosomal damage, impaired proteostasis and defective repair of compromised lysosomes leading to intraneuronal protein deposition and secretion of inflammatory cytokines. Our findings may lead to potential therapeutic strategies against ageing and AD.

## Results

### Ageing and proteostasis signatures in fibroblasts and tNeurons

**Fig. 1:**
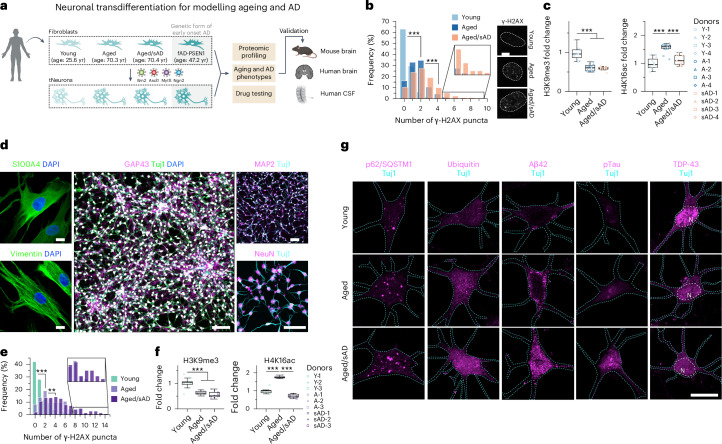
Transdifferentiation of human adult fibroblasts into neurons reveals signatures of ageing and AD. **a**, Human dermal fibroblasts were collected from healthy young and aged, aged/sAD and fAD-PSEN1 donors. Fibroblasts and tNeurons were used for a variety of experiments and the findings were validated in post-mortem brain tissue as well as CSF. **b**, Levels of DNA damage (left) measured as the number of the nuclear foci of γ-H2AX immunofluorescence (right) in human fibroblasts. White dotted lines outline cell nuclei. Images are representative of three independent experiments; *n* = 248 young, 304 aged and 252 aged/sAD cells from three donors. Scale bar, 50 μm. **c**, Age- and AD-related epigenetic alterations. Immunofluorescence of the histone modifications H3K9me3 (left) and H4K16ac (right) in human fibroblasts was measured. Data are from four donors and three independent experiments; H3K9me3, *n* = 252 young, 241 aged and 237 aged/sAD cells; H4K16ac, *n* = 157 young, 167 aged and 141 aged/sAD cells. **d**, Neuronal transdifferentiation efficiency. Representative images of human fibroblasts immunostained for S100A4 and Vimentin, and tNeurons immunostained for Tuj1, GAP43, MAP2 and NeuN with 4,6-diamidino-2-phenylindole (DAPI) counterstaining on PID 35. Scale bars, 100 μm. **e**, Analysis of DNA damage in tNeurons revealed by γ-H2AX immunofluorescence; *n* = 150 young, 132 (aged) and 141 (aged/sAD) cells. **b**,**e**, Inset: magnified views of the bars in the boxed regions. **f**, Immunofluorescence analysis of H3K9me3 and H4K16ac changes in tNeurons. H3K9me3, *n* = 95 young, 123 aged and 124 aged/sAD cells; H4K16ac, *n* = 112 young, 115 aged and 117 aged/sAD cells. **e**,**f**, Data are from three donors and three independent experiments. **g**, Representative images of proteostasis- and disease-associated protein markers—autophagy adaptor p62/SQSTM1, ubiquitin, Aβ42, pTau and TDP-43—in tNeurons. The cyan dashed line outlines tNeuron morphology determined by Tuj1 staining and the white dashed line represents the nuclear region (N). Scale bar, 20 μm. **c**,**f**, The boxes show the median and first and third quartiles (box boundaries), and the whiskers extend 1.5× the interquartile range from the boxes. Statistical analysis was performed using a one-way analysis of variance (ANOVA), followed by Bonferroni’s post-hoc analysis. **P < 0.01 and ***P < 0.001. Source numerical data are provided. Source data

We harnessed transcription factors—*Brn2*, *Ascl1*, *Myt1l* and *Ngn2*—along with small molecules to transdifferentiate human fibroblasts into cortical neurons (Fig. 1a). Fibroblasts were collected from eight healthy young (age, 25.6 ± 4.9 yr) and 12 aged donors (age, 70.3 ± 5.9 yr) as well as 16 aged patients with sAD (herein aged/sAD; age, 70.4 ± 9.2 yr). Fibroblasts from five middle-aged fAD patients carrying *PSEN1* mutations (herein fAD-PSEN1; age, 47.2 ± 10.2 yr) were subsequently derived for certain experiments (Supplementary Table 1). Fibroblasts from aged and aged/sAD donors showed an increase in DNA damage measured by γ-H2AX puncta (Fig. 1b) and a global loss of the epigenetic marker histone H3 Lys-9 trimethylation (H3K9me3; Fig. 1c). We observed histone H4 Lys-16 acetylation (H4K16ac) enriched during ageing but reduced with AD (Fig. 1c), recapitulating findings in human brain^22^.

**Extended Data Fig. 1:**
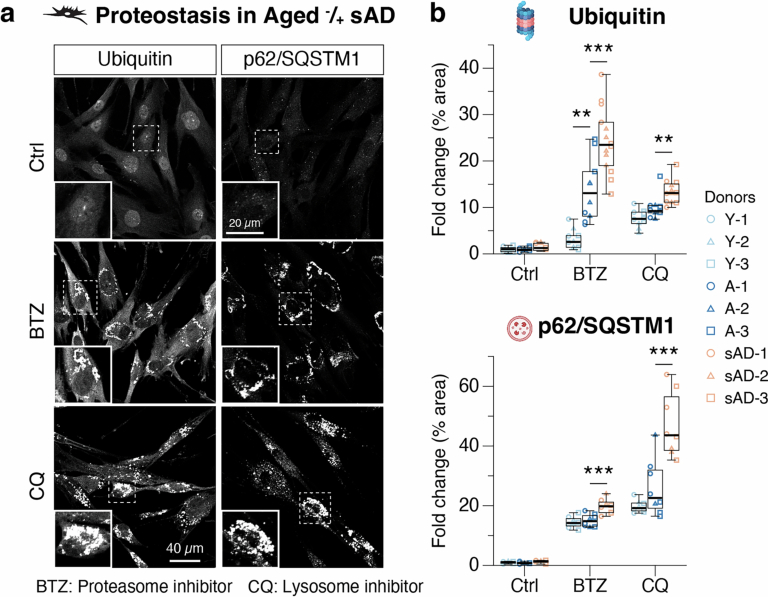
Proteostasis signatures of aging and Alzheimer’s disease (AD) are present in human adult fibroblasts. (**a**) Representative images of endogenous ubiquitin and p62/SQSTM1 in young fibroblasts treated with DMSO control (Ctrl), proteasome inhibitor Bortezomib (BTZ, 25 nM) or lysosome inhibitor Chloroquine (CQ, 25 µM) for 24 hr (scale bar: 40 μm). Insert: higher magnification view of ubiquitin and p62/SQSTM1 (scale bar: 20 μm). (**b**) Immunofluorescence (IF) quantification of proteostasis markers shown in panel (**a**) for changes in ubiquitin and p62/SQSTM1 by the treatment of BTZ and CQ, respectively, in young (Y), aged (A) and aged/sAD (sAD) fibroblasts. Data represent as fold changes in ubiquitin and p62/SQSTM1 levels (% of area per image field) relative to young fibroblasts treated with DMSO Ctrl. Ubiquitin: Ctrl: *n* = 230 (young), 215 (aged) and 162 (aged/sAD) cells; BTZ: *n* = 124 (young), 142 (aged) and 131 (aged/sAD) cells; CQ: *n* = 152 (young), 131 (aged) and 134 (aged/sAD) cells. p62/SQSTM1: Ctrl: *n* = 140 (young), 117 (aged) and 138 (aged/sAD) cells; BTZ: *n* = 155 (young), 130 (aged) and 143 (aged/sAD) cells; CQ: *n* = 133 (young), 150 (aged) and 137 (aged/sAD) cells. In panel **b**, the boxes show median and 1^st^ and 3^rd^ quartile and the whiskers extending 1.5 times the interquartile range from the boxes. Data show box-and-whisker plots of three to five independent experiments and cells from three independent healthy control (HC) and AD donors. Statistical analysis is performed using Two-Way ANOVA followed by Bonferroni post-hoc analysis. **P < 0.01 and ***P < 0.001. Source numerical data are provided. Source data

We next assessed defects in global proteostasis state by monitoring the formation of ubiquitin-positive (Ub^+^) and autophagy receptor p62/SQSTM1 puncta. There were no obvious Ub^+^ or p62/SQSTM1 puncta in the fibroblasts under basal conditions. When exposed to proteotoxic stress, that is, sublethal dosages of the proteasome inhibitor bortezomib or the lysosome inhibitor chloroquine, fibroblasts from aged donors showed a moderate increase in an accumulation of Ub^+^ and p62/SQSTM1, and a much higher increase in those of aged/sAD donors (Extended Data Fig. 1a,b). In agreement with previous findings in human brain^22–24^, our results demonstrated a correlation between the proteostasis vulnerability of fibroblasts with the age and disease status of the donor.

**Extended Data Fig. 2:**
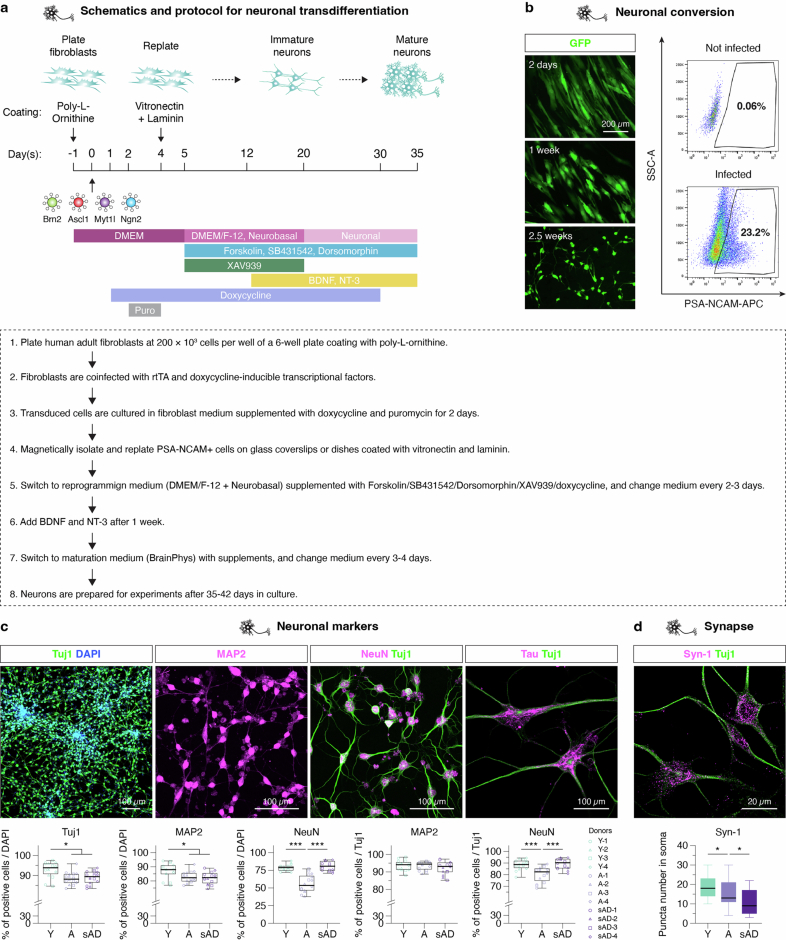
Cortical neurons are generated directly from human adult fibroblasts using a combinatorial transcription-factor and small-molecule protocol. (**a**) Transdifferentiation scheme using transcription factors *Brn2*, *Ascl1*, *Myt1l*, and *Ngn2* (herein BAMN factors) that induces a global transcriptional change in fibroblasts to constitute a new neuronal cell state. Fibroblasts are plated one day before transdifferentiation. On Day 0, cells are infected with lentiviruses expressing BAMN factors and harvested four days after doxycycline-induction. Transduced cells expressing PSA-NCAM are magnetically isolated and replated to vitronectin/laminin-coated plates on Day 4 and then switched to reprogramming medium (DMEM/F12/Neurobasal) supplemented with small molecules 5 µM Forskolin, 10 µM SB 431542, 2 µM Dorsomorphin and 2 µM XAV939 on Day 5. 10 ng/mL BDNF and NT-3 are added into the reprogramming medium after one week. Cells are switched to maturation medium (Neuronal) on Day 20 and used for experiments after Day 35. (**b**) Fibroblasts are infected with lentiviruses expressing BAMN factors and GFP and monitored for morphological changes after lentiviral induction. Transduced cells undergo sorting based on PSA-NCAM+ gate in flow cytometry on Day 4. Scale bar: 200 μm. (**c**,**d**) IF images of neuronal markers in tNeurons at PID 35. Quantification of the percentage of cells positive for both NeuN and Tuj1 (**c**) and the numbers of Syn-1 puncta in the soma (**d**). Tuj1/DAPI, MAP2/DAPI & MAP2/Tuj1: *n* = 1208 (young), 678 (aged) and 789 (aged/sAD) cells; NeuN/DAPI and NeuN/Tuj1: *n* = 822 (young), 579 (aged) and 759 (aged/sAD) cells. Syn-1: *n* = 40 (young), 42 (aged) and 41 (aged/sAD) cells. Scale bars: 100 μm (**c**) and 20 μm (**d**). In panels **c** and **d**, the boxes show median and 1^st^ and 3^rd^ quartile and the whiskers extending 1.5 times the interquartile range from the boxes. Data show box-and-whisker plots of two to four independent experiments and cells from independent HC and AD patients. Statistical analysis is performed using One-Way ANOVA followed by Bonferroni post-hoc analysis. *P < 0.05 and ***P < 0.001. Source numerical data are provided. Source data

**Extended Data Fig. 3:**
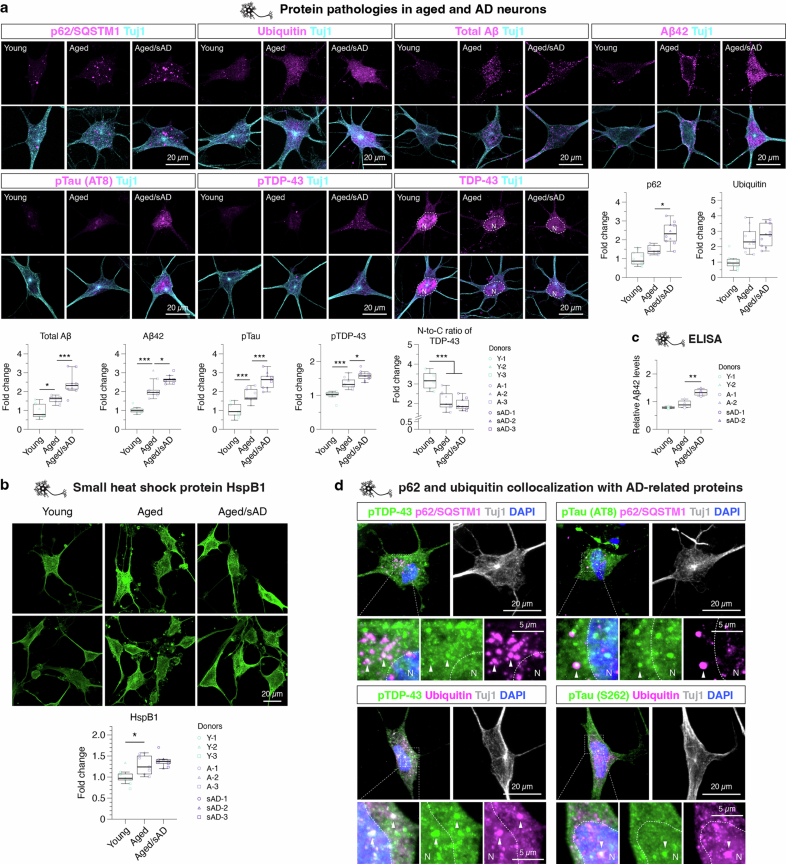
Human tNeurons derived from sAD patients exhibit disease-related protein pathologies. (**a**) IF images and quantification of proteostasis- and disease-associated protein markers, including p62/SQSTM1, ubiquitin, total Aβ and the toxic isoform Aβ42, hyperphosphorylated tau (pTau) and TDP-43 (pTDP-43) and nuclear-to-cytoplasmic (N-to-C) ratio of TDP-43 in tNeurons shown as full images for Fig. 1g. Cells are co-stained with Tuj1. White dash line represents the nuclear region (N). p62/SQSTM1: *n* = 62 (young), 62 (aged) and 66 (aged/sAD) cells; ubiquitin: *n* = 46 (young), 53 (aged) and 66 (aged/sAD) cells; total Aβ: *n* = 111 (young), 106 (aged) and 114 (aged/sAD) cells; Aβ42: *n* = 103 (young), 95 (aged) and 93 (aged/sAD) cells; pTau: *n* = 91 (young), 89 (aged) and 114 (aged/sAD) cells; pTDP-43: *n* = 91 (young), 74 (aged) and 95 (aged/sAD) cells; N-to-C ratio of TDP-43: *n* = 85 (young), 86 (aged) and 103 (aged/sAD) cells. Scale bar: 20 μm. (**b**) IF staining and quantification of small heat shock protein HspB1 in tNeurons. *n* = 80 (young), 111 (aged) and 134 (aged/sAD) cells. Scale bar: 20 μm. (**c**) Sandwich ELISA assay for detecting endogenous Aβ42 in total cell lysates of tNeurons. The values are revealed by a fold change relative to young tNeurons. *n* = 4 (young), 4 (aged) and 4 (aged/sAD) independent replicates. (**d**) IF staining of pTau and pTDP-43 with p62/SQSTM1 and ubiquitin in aged/sAD tNeurons (scale bar: 20 μm). Cells are co-stained with Tuj1 and DAPI. White dash line represents the nuclear region (N). Insert: higher magnification view of protein co-localization within individual neuron (scale bar: 5 μm). Arrowhead: protein co-localization. In panels **a**, **b** and **c**, the boxes show median and 1^st^ and 3^rd^ quartile and the whiskers extending 1.5 times the interquartile range from the boxes. Data show box-and-whisker plots of two to three independent experiments and cells from independent HC and AD patients. Statistical analysis is performed using One-Way ANOVA followed by Bonferroni post-hoc analysis. *P < 0.05, **P < 0.01 and ***P < 0.001. Source numerical data are provided. Source data

We next generated tNeurons on post-induction days (PID) 35–42 (Fig. 1d and Extended Data Fig. 2a,b). Overall, our protocol can efficiently transdifferentiate fibroblasts, with a slight reduction in the fibroblast-to-neuron conversion efficiency in aged donors (Extended Data Fig. 2c,d). The tNeurons retained epigenetic hallmarks (Fig. 1e,f). Surprisingly, the proteostasis deficits were exhibited under basal conditions in aged and AD tNeurons unlike what was observed in the originating fibroblasts. Thus, Ub^+^ and p62/SQSTM1 puncta were robustly increased in aged/sAD tNeurons (Fig. 1g and Extended Data Fig. 3a). To assess whether aged and AD tNeurons exhibit constitutive deficits in other proteostasis pathways, we monitored the levels of the small heat shock protein HspB1 (Extended Data Fig. 3b). HspB1 levels increased in aged and aged/AD tNeurons in comparison to young tNeurons. These experiments indicate that fibroblasts and the derived neurons retain hallmarks of ageing and sAD, and that age- and AD-dependent proteostasis deficits become exacerbated in neurons.

### Proteotoxic inclusions formed in aged/sAD tNeurons

We next examined AD-associated protein pathologies in tNeurons. We found dramatic increases in deposits of intracellular total Aβ and toxic isoform Aβ42 as well as hyperphosphorylated tau (pTau) in aged/sAD tNeurons (Fig. 1g and Extended Data Fig. 3a). Increased Aβ42 levels in the lysates of aged/sAD tNeurons were confirmed by a sensitive ELISA detection assay (Extended Data Fig. 3c). TDP-43 deposits are a pathological hallmark of amyotrophic lateral sclerosis and frontotemporal dementia^25^, but occur in 23–50% of AD cases^26,27^. We also observed increased TDP-43 pathology in aged/sAD tNeurons, including cytoplasmic mislocalization of nuclear TDP-43 and hyperphosphorylated TDP-43 (pTDP-43) (Fig. 1g and Extended Data Fig. 3a). pTau inclusions partially co-localized with p62/SQSTM1 puncta, whereas pTDP-43 partially co-localized with Ub^+^ puncta (Extended Data Fig. 3d). Together, these experiments indicate that deficits in neuronal proteostasis are exacerbated by ageing and AD, cooperatively promoting the cell-intrinsic accumulation of multiple AD-related protein pathologies.

### Quantitative proteomics of young, aged and aged/sAD tNeurons

**Fig. 2:**
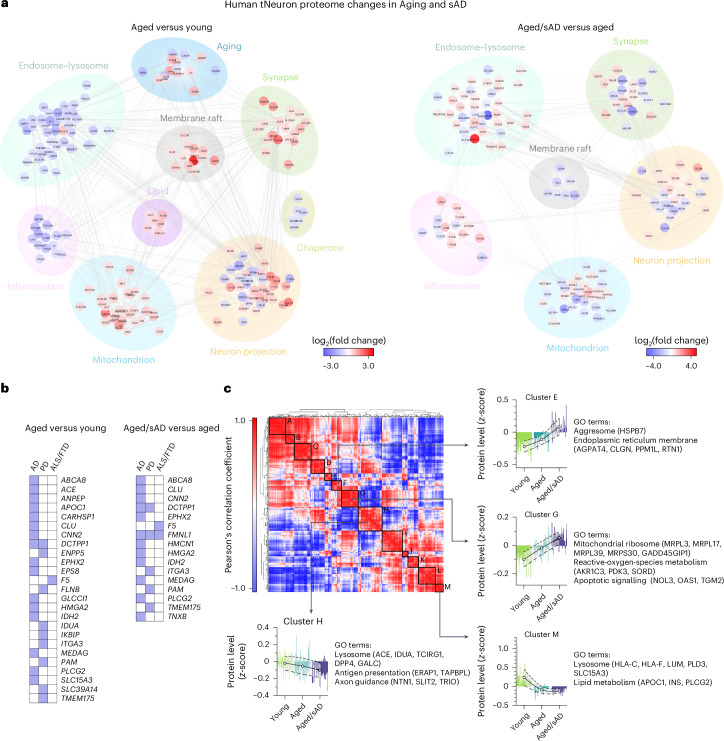
Human tNeurons carry proteomic signatures of ageing and AD. **a**, Differential expression of proteins detected in tNeurons of healthy young (*n* = 3) and aged (*n* = 3) individuals as well as patients with aged/sAD (*n* = 6) on PID 40. The top pathways for ageing and sAD proteomes were analysed using gene ontology databases. Comparisons between tNeurons from aged and young donors (left) as well as aged/sAD and aged donors (right). The level of enrichment of the identified proteins (log_2_-transformed fold change) is represented by coloured circles (increase in red and decrease in blue). **b**, List of differentially expressed proteins associated with risk genes for age-related neurodegenerative diseases. PD, Parkinson’s disease; ALS/FTD, amyotrophic lateral sclerosis/frontotemporal dementia. **c**, Cluster heatmap of the Pearson’s correlation coefficients of total tNeuron protein expression (top left). Clusters A to M (right and bottom) show distinct protein expression patterns and the associated gene ontology terms between the young, age and aged/sAD samples. Each line represents the expression of individual protein defined by the relative protein abundance (*z*-score) across different groups. The white circles represent the average *z*-score for each cluster and the dashed lines represent the s.d.

**Extended Data Fig. 4:**
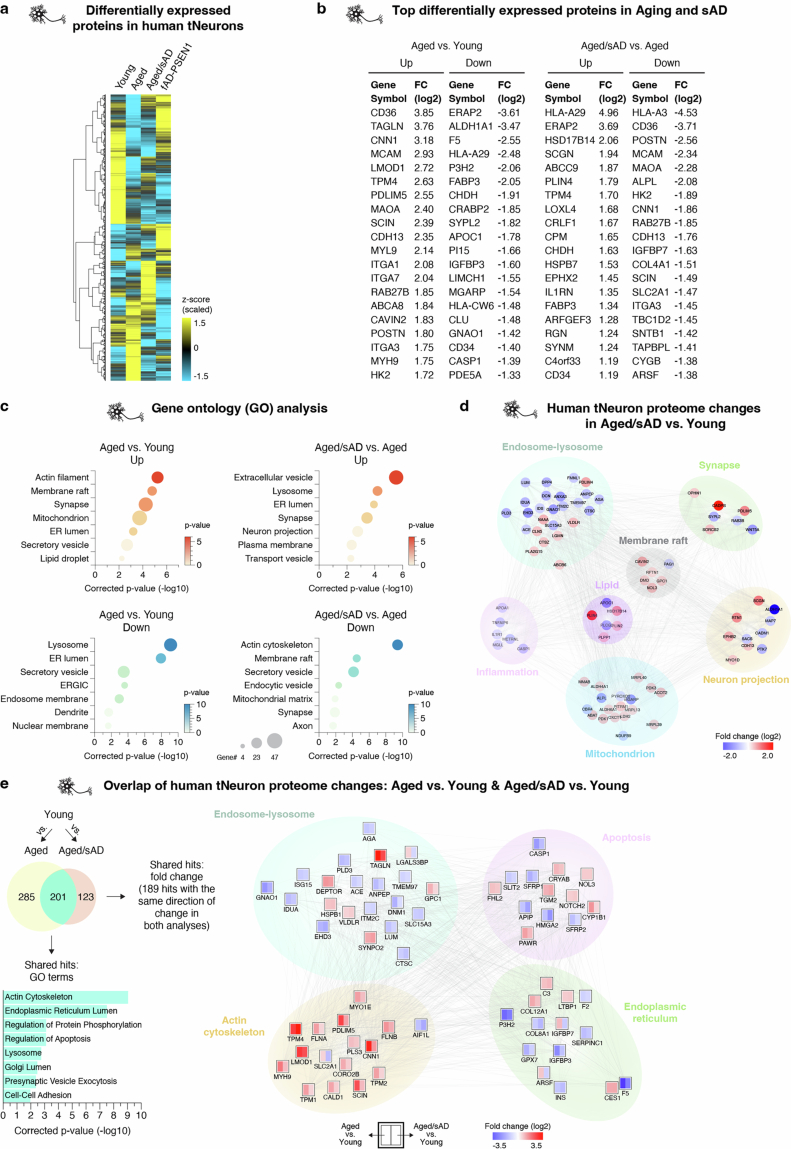
Quantitative proteomic analysis of human tNeurons identifies proteins and pathways associated with aging and sAD. (**a**) Heatmap of the differentially expressed proteins across tNeurons from young, aged, aged/sAD and fAD-PSEN1 donors. (**b**) Top-ranked proteins (rows) changing with aging and sAD (columns) based on log2-fold change (log2-FC). (**c**) Gene ontology (GO) analysis of the differentially expressed proteins between young, aged and aged/sAD tNeurons. Circle sizes reflect the number of proteins. (**d**) Network analysis of tNeuron proteomes, including proteins in the top-ranked pathways associated with young and sAD, revealed by GeneMANIA and GO. Comparison between healthy young (*n* = 3 individuals) and aged/sAD tNeurons (*n* = 6 individuals) at PID 40. Coloured circles represent the enrichment of identified proteins revealing by log2-FC: increase in red and decrease in blue. (**e**) Venn diagram reveals an overlap of differentially expressed proteins between tNeurons from aged and young donors, and between tNeurons from aged/sAD and young donors. The shared hits and assigned GO terms are showed in light green colour. Interaction network for the shared hits that are either increased or decreased in aged and aged/sAD tNeurons as compared with young tNeurons. Each node representing a single protein that is divided to reflect the individual change for aged vs. young (left) and aged/sAD vs. young (right). Protein abundance increases are shown in red, and decreases shown in blue.

The transcriptome of the neurons of patients with AD has been extensively characterized but often does not reflect the state of their proteome^28–30^. Accordingly, we carried out quantitative proteomic analyses of young, aged and aged/sAD tNeurons. Notably, the top-ranked pathways altered by ageing and AD included proteostasis and organelle homeostasis (Fig. 2a and Extended Data Fig. 4a–c). We observed age-related upregulation of proteins modulating mitochondria and synapse, whereas proteins in the endosome–lysosomal pathway (for example, CLU, CTSC and TMEM175) were mostly downregulated with ageing. Comparisons of the changes in aged and aged/sAD tNeurons relative to young tNeurons revealed shared aged and aged/sAD protein hits, with over 94% of them showing the same direction of expression changes in both aged and aged/sAD tNeurons (Extended Data Fig. 4d,e). A comparison of aged/sAD and aged proteomic changes revealed sAD-specific changes, which included upregulation of proteins annotated as endosome–lysosome (for example, CLU), mitochondria (for example, CHDH), inflammation (for example, PYCARD) and synapse (for example, SORCS2) as well as sAD-specific downregulation of membrane and vesicular trafficking proteins (Fig. 2a). Remarkably, many aged/sAD proteome changes corresponded to proteins encoded by genes associated with risk of neurodegenerative diseases (for example, AD, PD and frontotemporal dementia; Fig. 2b), mostly involved in the endosome–lysosomal pathway.

Cluster analysis of our proteomic data based on the similarity of protein expression across young, aged and aged/sAD tNeurons led to the identification of related subsets of proteins with unique trajectories of change during ageing and AD (Fig. 2c). Clusters E and G exhibited increased protein expression going from young to aged to aged/sAD tNeurons. In contrast, clusters H and M exhibited gradual decreased protein expression. These clusters included proteins regulating lysosome and lipid metabolism. The endosome–lysosomal system indeed seems to be a major pathway affected by ageing and AD, with many changed proteins associated with lysosomes, notably proteins involved in lysosomal quality control (LQC) were altered in ageing (for example, CNN2 and HspB1) and AD (for example, DPP7, PLBD2, PLD3 and TAGLN).

### PSEN1 mutations elicit pathological and proteome alterations

**Extended Data Fig. 5:**
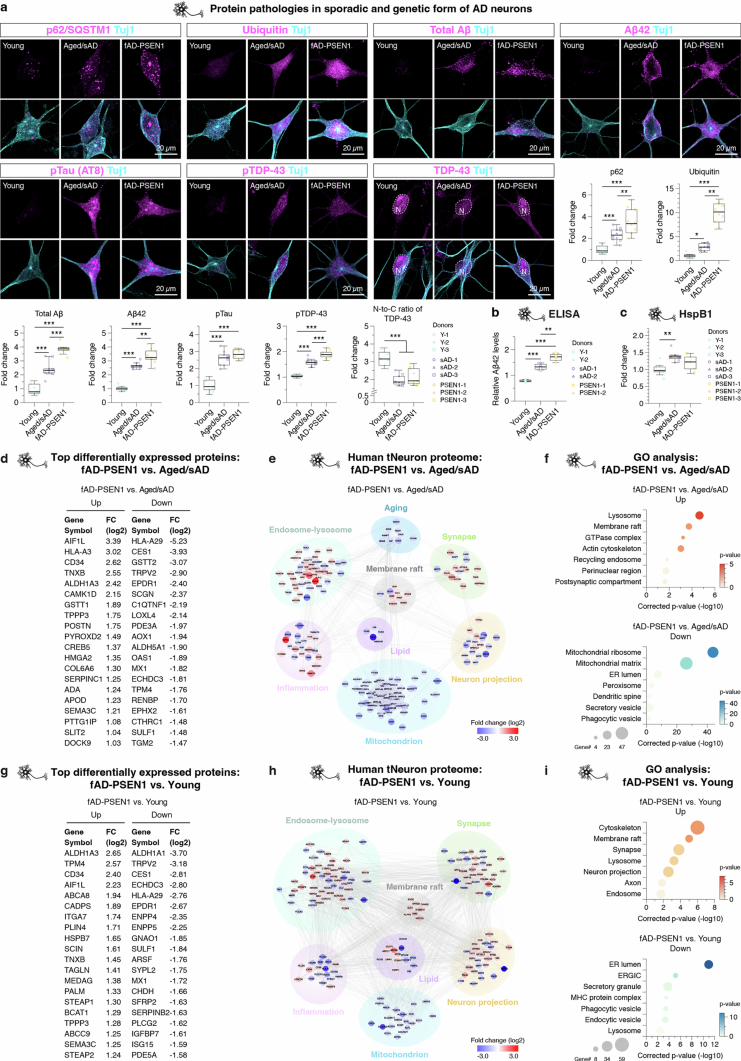
Quantitative proteomic and molecular analysis of tNeurons identify signatures of pathogenic mutations in the *PSEN1* gene in AD patient neurons. (**a**) IF images and quantification of proteostasis- and disease-associated protein markers in tNeurons from healthy young donors as well as AD patients with aged/sAD and fAD-PSEN1. White dash line represents the nuclear region (N). p62/SQSTM1: *n* = 62 (young), 66 (aged/sAD) and 49 (fAD-PSEN1) cells; ubiquitin: *n* = 46 (young), 66 (aged/sAD) and 50 (fAD-PSEN1) cells; total Aβ: *n* = 111 (young), 114 (aged/sAD) and 57 (fAD-PSEN1) cells; Aβ42: *n* = 103 (young), 93 (aged/sAD) and 68 (fAD-PSEN1) cells; pTau: *n* = 91 (young), 114 (aged/sAD) and 107 (fAD-PSEN1) cells; pTDP-43: *n* = 91 (young), 95 (aged/sAD) and 82 (fAD-PSEN1) cells; N-to-C ratio of TDP-43: *n* = 85 (young), 103 (aged/sAD) and 109 (fAD-PSEN1) cells. Scale bar: 20 μm. (**b**) Detection of endogenous Aβ42 in total cell lysates of tNeurons using sandwich ELISA assay. The values are revealed by a fold change relative to young tNeurons. *n* = 4 (young), 4 (aged/sAD) and 4 (fAD-PSEN1) independent replicates. (**c**) IF quantification of small heat shock protein HspB1 in young, aged/sAD and fAD-PSEN1 tNeurons. *n* = 80 (young), 134 (aged/sAD) and 136 (fAD-PSEN1) cells. (**d**) Top-ranked proteins (rows) changing with aged/sAD and fAD-PSEN1 (columns) based on log2-FC. (**e**) Network analysis of tNeuron proteins in the top-ranked pathways associated with aged/sAD and fAD-PSEN1. Comparison between aged/sAD (*n* = 6 individuals) and fAD-PSEN1 (*n* = 2 individuals) at PID 40. Coloured circles represent the enrichment of identified proteins revealing by log_2_FC: increase in red and decrease in blue. (**f**) GO analysis of the differentially expressed proteins across aged/sAD and fAD-PSEN1 tNeurons. Circle sizes reflect the number of proteins. (**g**) Lists of top-ranked proteins (rows) changing with fAD-PSEN1 and young (columns) based on log_2_FC. (**h**) Network analysis and differential expression of proteins detected in tNeurons from healthy young donors (*n* = 3 individuals) and patients with fAD-PSEN1 (*n* = 2 individuals) at PID 40. The top-ranked pathways for fAD-PSEN1 proteome are analysed using GO databases. Coloured circles represent the enrichment of identified proteins revealing by log_2_FC: increase in red and decrease in blue. (**i**) GO analysis of the differentially expressed proteins across fAD-PSEN1 and young tNeurons. Circle sizes reflect the number of proteins. In panels **a**, **b** and **c**, the boxes show median and 1^st^ and 3^rd^ quartile and the whiskers extending 1.5 times the interquartile range from the boxes. Data show box-and-whisker plots of two to three independent experiments and cells from independent HC and AD patients. Statistical analysis is performed using One-Way ANOVA followed by Bonferroni post-hoc analysis. *P < 0.05, **P < 0.01 and ***P < 0.001. Source numerical data are provided. Source data

We next examined tNeurons derived from fibroblasts of fAD-PSEN1 donors. *PSEN1* mutants elicit early development of AD through increased production of Aβ42 (ref. ^31^). The fAD-PSEN1 tNeurons obtained from middle-aged donors also manifested basal accumulations of Ub^+^ and p62/SQSTM1 and contained comparable or higher levels of Aβ, pTau, p-TDP-43 and HspB1 than aged/sAD tNeurons (Extended Data Fig. 5a–c). We next compared the proteomic analysis of fAD-PSEN1 tNeurons with the proteomes of either aged/sAD tNeurons (Extended Data Fig. 5d–f) or young tNeurons (Extended Data Fig. 5g–i) to identify top candidate hits and common top-ranked pathways. Consistent with the younger age of the donors, the fAD-PSEN1 tNeurons had reduced expression of proteins in the ‘ageing’ category (Extended Data Fig. 5e). The most dramatic change in fAD-PSEN1 tNeurons was the downregulation of mitochondrial proteins.

### Constitutive lysosomal damage augmented in aged/sAD tNeurons

**Fig. 3:**
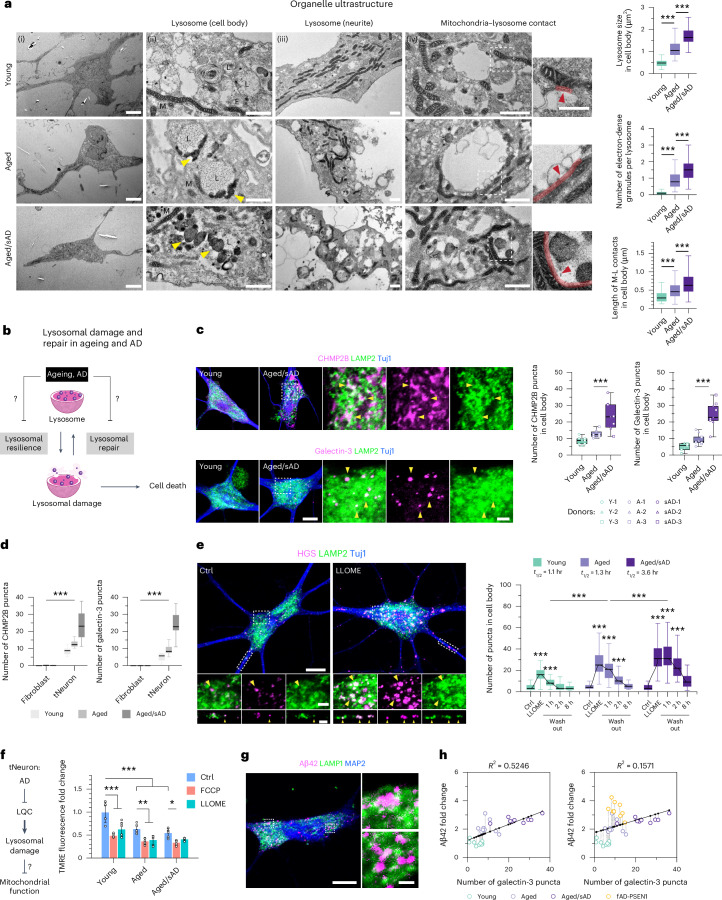
Constitutive lysosomal damage and lysosomal repair deficits in AD tNeurons. **a**, Analysis of the organelle ultrastructure of human tNeurons using TEM (left). E, endosome; L, lysosome; M, mitochondria. Yellow arrowheads point to electron-dense granules and red arrowheads and lines indicate the mitochondria–lysosome contact site. Insets: higher magnification views of the regions in the white boxes showing mitochondria–lysosome contact. The lysosome size (top right; *n* = 59 young, 69 aged and 69 aged/sAD lysosomes), electron-dense material abundance (middle right; *n* = 74 young, 68 aged and 60 aged/sAD lysosomes) and length of mitochondria–lysosome contacts (bottom right; *n* = 174 young, 262 aged and 246 aged/sAD contacts) from two donors and two independent experiments were determined. Scale bars, 10 μm (i), 1 μm (ii–iv) and 500 nm (insets). **b**, Schematic of the tests to determine how ageing and AD alters lysosomal damage responses, leading to cell death. **c**, Lysosomal damage under basal conditions. Representative images of tNeurons under basal conditions immunostained on PID 35 for LAMP2, Tuj1 and ESCRT-III CHMP2B or galectin-3 (left). Insets: higher magnification views of the region in the white boxes showing protein co-localization. The yellow arrowheads point to CHMP2B and galectin-3 co-localization with LAMP2. Numbers of CHMP2B and galectin-3 puncta (immunofluorescence quantification) in the cell body of tNeurons (right). CHMP2B, *n* = 117 young, 103 aged and 97 aged/sAD cells; galectin-3, *n* = 111 young, 108 aged and 95 aged/sAD cells. Scale bars, 10 μm (main images; first two columns on the left) and 2 μm (insets). **d**, Basal state lysosomal damage. Comparison of the numbers of CHMP2B and galectin-3 puncta in fibroblasts and tNeurons at basal conditions. Fibroblasts: CHMP2B, *n* = 102 young, 105 aged and 99 aged/sAD cells; galectin-3, *n* = 102 young, 105 aged and 99 aged/sAD cells. tNeurons: CHMP2B, *n* = 117 young, 103 aged and 97 aged/sAD cells; galectin-3, *n* = 111 young, 108 aged and 95 aged/sAD cells. **e**, Lysosomal repair following lysosomal damage. Representative images of AD tNeurons immunostained on PID 36 for LAMP2, ESCRT-0 HGS protein and Tuj1. Insets: magnified views of the regions in the white boxes (middle and bottom left). The yellow arrowheads point to HGS co-localization with LAMP2. Scale bars, 10 μm (main images) and 2 μm (insets). Cells were treated with 0.25 mM LLOME for 30 min, followed by LLOME washout for lysosomal repair. Numbers of HGS puncta in the cell body determined from the immunofluorescence images (right). The time required for lysosomal repair is indicated (*t*_1/2_). Young, *n* = 108 control (Ctrl), 107 LLOME, 111 washout 1 h, 118 washout 2 h and 90 washout 8 h cells; aged, *n* = 110 Ctrl, 113 LLOME, 114 washout 1 h, 116 washout 2 h and 109 washout 8 h cells; aged/sAD, *n* = 115 Ctrl, 115 LLOME, 117 washout 1 h, 109 washout 2 h and 79 washout 8 h cells. **c**–**e**, Data are from three donors and three independent experiments. **f**, Schematic of the tests to determine whether defective LQC mediates mitochondrial dysfunction in ageing and AD. The mitochondrial membrane potential (fold change relative to young tNeurons treated with dimethylsulfoxide, DMSO) was quantified using TMRE staining following treatment with DMSO (Ctrl), 20 µM FCCP or 0.25 mM LLOME for 30 min. Data are the mean ± s.d.; *n* = 6 independent replicates (all groups) from three donors and two experiments. **g**, Aβ42 deposits in aged/sAD lysosomes. Immunofluorescence analysis of co-localization of Aβ42 with LAMP1 in aged/sAD tNeurons. Inset: higher magnification views of the regions in the white boxes showing Aβ42 and LAMP1. Scale bars, 10 μm (main image) and 1 μm (insets). **h**, Correlation between intracellular Aβ42 levels and galectin-3 puncta numbers, indicating lysosomal damage, in different groups of tNeurons. The black line represents the fitted linear correlation; Pearson’s correlations were used to calculate the *R*^2^ values; *n* = 9 independent replicates from three donors and three experiments. **a**,**c**–**e**, The boxes show the median and first and third quartiles (box boundaries), and the whiskers extend 1.5× the interquartile range from the boxes. **a**,**c**–**f**, Statistical analyses were performed using a one-way (**a**,**c**) or two-way (**d**–**f**) ANOVA, followed by Bonferroni’s post-hoc analysis. *P < 0.05, **P < 0.01 and ***P < 0.001. Source numerical data are provided. Source data

Given the changes in the lysosomal proteome in aged and aged/sAD tNeurons, we used transmission electron microscopy (TEM) to characterize lysosomal ultrastructure (Fig. 3a). Comparisons of young, aged and aged/sAD tNeurons revealed progressive increases in the size of individual lysosomes and increased presence of electron-dense granules. Small electron-dense granules were specifically found proximal to the lysosomal membrane in aged tNeurons, whereas large dense granules accumulated within the lysosomes of aged/sAD tNeurons. Notably, aged and aged/sAD tNeurons contained mitochondria closely surrounding the enlarged lysosomes in the cell body (red arrow in Fig. 3a (right)). Quantification provided support for an increase in mitochondria–lysosome contacts in AD neurons.

**Extended Data Fig. 6:**
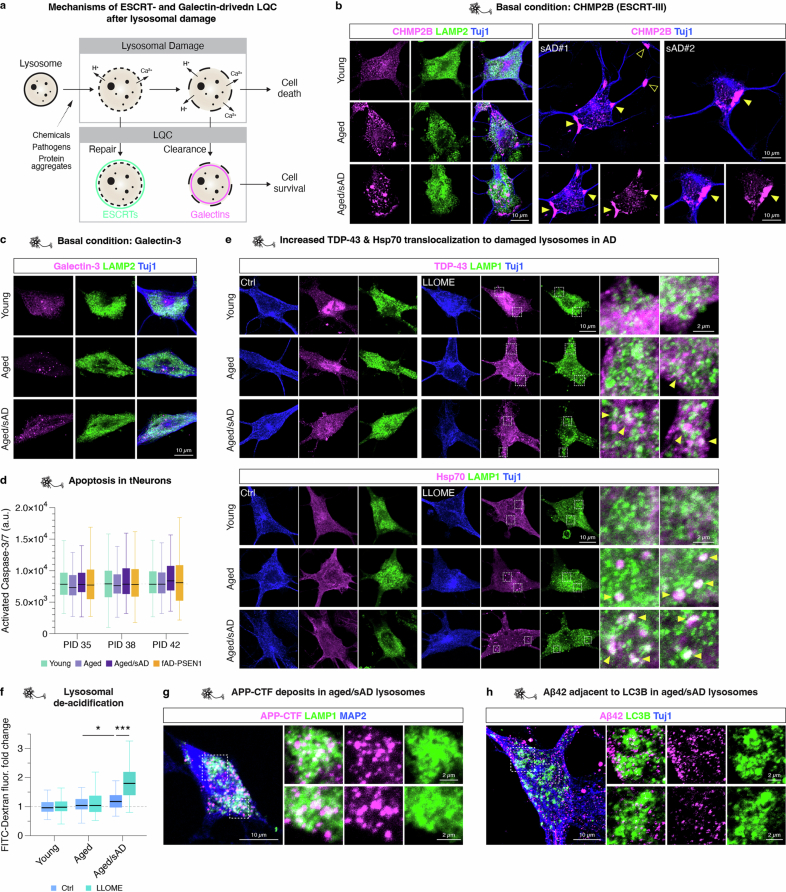
Aberrant response to lysosomal damage at basal conditions and during LLOME treatment are prominent in aged/sAD tNeurons. (**a**) Schematic for describing the ESCRT- and Galectin-mediated lysosomal quality control (LQC) machinery. When undergoing lysosomal damage stress, lysosomal membrane is subjected to rupture. To avoid the leakage of lysosomal contents and activation of cell death pathways, the compromised lysosomes recruit ESCRT proteins (ESCRTs) to repair the small membrane wounds and Galectins to target the massively damaged lysosomes for lysophagy. (**b**) Representative images for immunostaining of CHMP2B (magenta), LAMP2 (green) and Tuj1 (blue) in young, aged and aged/sAD tNeurons, related to Fig. 3c. Arrowhead: high-density accumulations of CHMP2B in the vicinity of plasma membrane (solid) and within neurites (hollow). Scale bar: 10 μm. (**c**) Representative images for immunostaining of Galectin-3 (magenta), LAMP2 (green) and Tuj1 (blue) in young, aged and aged/sAD tNeurons, related to Fig. 3c. Scale bar: 10 μm. (**d**) Measurement of spontaneous cell death, indicated by apoptosis markers (for example active Caspase-3/7), in young, aged, aged/sAD and fAD-PSEN1 tNeurons during in vitro culture at PID 35, 38 and 42. PID 35: *n* = 162 (young), 158 (aged), 148 (aged/sAD) and 136 (fAD-PSEN1) cells; PID 38: *n* = 163 (young), 159 (aged), 147 (aged/sAD) and 156 (fAD-PSEN1) cells; PID 42: *n* = 159 (young), 151 (aged), 142 (aged/sAD) and 145 (fAD-PSEN1) cells. (**e**) Immunostaining of TDP-43 and Hsp70 (magenta), LAMP1 (green) and Tuj1 (blue) during DMSO Ctrl or 0.25 mM LLOME treatment for 30 min in young, aged, aged/sAD tNeurons at PID 35 (scale bar: 10 μm). Insert: higher magnification view of protein co-localization within individual neuron (scale bar: 2 μm). Arrowhead: protein co-localization. (**f**) Changes in lysosomal acidification by detecting the intensity of preloaded pH-sensitive FITC-conjugated Dextran in tNeurons at basal conditions and during 0.25 mM LLOME treatment for 30 min. Ctrl: *n* = 145 (young), 135 (aged) and 148 (aged/sAD) cells; LLOME: *n* = 142 (young), 137 (aged) and 155 (aged/sAD) cells. (**g**) IF analysis of co-localization of APP-CTF with LAMP1 in aged/sAD tNeurons (scale bar: 10 μm). Insert: higher magnification view of APP-CTF and LAMP1 (scale bar: 2 μm). (**h**) IF analysis of co-localization of Aβ42 with LC3B in aged/sAD tNeurons (scale bar: 10 μm). Insert: higher magnification view of Aβ42 and LC3B (scale bar: 2 μm). In panels **d** and **f**, the boxes show median and 1^st^ and 3^rd^ quartile and the whiskers extending 1.5 times the interquartile range from the boxes. Data show box-and-whisker plots of three independent experiments and cell lines from independent HC and AD donors. Statistical analysis is performed using Two-Way ANOVA followed by Bonferroni post-hoc analysis. Source numerical data are provided. Source data

Based on our proteomic and TEM analyses, we hypothesized that the cellular state of aged and AD tNeurons affects lysosomal resilience to damage and/or restoration from damage (Fig. 3b). Complex LQC machineries recognize damaged lysosomes to facilitate either repair or clearance and protect cells against lysosomal membrane permeabilization and cell death^32–35^. Two well-characterized mechanisms involve endosomal sorting complex required for transport proteins (ESCRTs) and galectins, which target mildly and severely damaged lysosomes, respectively (Extended Data Fig. 6a). To assess lysosomal integrity under basal conditions, we first measured the constitutive recruitment of ESCRTs and galectins to lysosomes. We detected no appreciable levels of damaged lysosomes in young tNeurons, a slight increase in the number and intensity of ESCRT-III CHMP2B- and galectin-3-containing puncta in aged tNeurons, and a dramatic increase in these lysosomal damage markers in aged/sAD tNeurons (Fig. 3c and Extended Data Fig. 6b,c). We also found aberrant accumulations of CHMP2B adjacent to the plasma membrane and neurite branch points (Extended Data Fig. 6b), providing further evidence for the participation of CHMP2B in both plasma and lysosomal membrane repair^34–36^. Nonetheless, no spontaneously apoptotic death was observed in any of the tNeuron groups, as indicated by the levels of activated caspase-3 and -7 (caspase-3/7; Extended Data Fig. 6d). These results indicate that aged/sAD tNeurons carry a substantial burden of constitutively damaged lysosomes. Interestingly, similar analyses in the parental fibroblasts did not reveal obvious CHMP2B^+^ or galectin-3^+^ puncta for any donor groups under basal conditions, in contrast to what we observed in tNeurons (Fig. 3d).

### Ageing and sAD impact neuronal lysosomal repair pathways

We next examined whether the lysosomal repair dynamics are impaired in aged and aged/sAD tNeurons. Thus, constitutive lysosomal damage in aged and aged/sAD tNeurons could overwhelm the LQC machineries and limit cellular capacity to counter additional lysosomal stress. Lysosomal damage was induced by a 30-min incubation with a well-validated lysosomotropic reagent, l-leucyl-l-leucine *O*-methyl ester (LLOME)^37–39^, followed by a chase for up to 8 h after LLOME washout to assess repair kinetics. Given the robust baseline presence of ESCRT-III CHMP2B puncta in aged/sAD tNeurons, we chose ESCRT-0 HGS to monitor the spatiotemporal change of the lysosomal damage response because its baseline distribution was comparable across all tNeuron groups (Fig. 3e). Lysosomal damage caused by LLOME treatment indeed increased the number of HGS puncta; when compared with young tNeurons, aged tNeurons showed a moderate increase and aged/sAD tNeurons showed a substantial increase. Following LLOME washout, the half-life estimates (*t*_1/2_) of HGS puncta decrease revealed differences in the efficiency of lysosomal repair. In young tNeurons, HGS puncta rapidly returned to baseline levels with a *t*_1/2_ of about 1 h. However, aged tNeurons exhibited a slight delay and aged/sAD tNeurons exhibited a threefold delay (Fig. 3e). These experiments indicate that lysosomal repair pathways are progressively impaired in aged and aged/sAD tNeurons.

### Lysosomal damage impacts other proteostasis pathways

Lysosomal damage and repair have been linked to several proteostasis pathways^40–42^ as well as RNA-containing stress granules^32^. We thus examined how LLOME-induced damage affects the RNA-binding protein TDP-43 and the molecular chaperone Hsp70 in the different tNeuron classes. Although cytoplasmic TDP-43 was basally increased in aged/sAD tNeurons (Fig. 1g), we observed that TDP-43 mislocalized to damaged lysosomes, particularly in LLOME-treated aged/sAD tNeurons (Extended Data Fig. 6e). Cytoplasmic Hsp70 was basally diffuse in all tNeuron classes and remained diffuse in LLOME-treated young tNeurons but was strongly recruited to damaged lysosomes in aged/sAD tNeurons (Extended Data Fig. 6e). These experiments suggest that lysosomal damage probably reverberates through the network, affecting other proteostasis processes.

Because aged/sAD tNeurons have altered mitochondrial proteomes and increased mitochondria–lysosome contacts, we considered a potential interplay between lysosomal damage and mitochondrial dysfunction in AD. We used tetramethylrhodamine ethyl ester (TMRE), which accumulates only in metabolically active mitochondria. As expected, TMRE fluorescence was reduced in tNeurons treated with an uncoupler of the mitochondrial respiratory chain, carbonyl cyanide 4-(trifluoromethoxy)phenylhydrazone (FCCP; Fig. 3f). Under basal conditions, TMRE fluorescence was lower in aged and aged/sAD tNeurons compared with young tNeurons, which is indicative of basal mitochondrial impairment. Lysosomal damage with a 30-min LLOME treatment also led to decreased TMRE fluorescence intensity in all tNeuron classes, revealing a link between lysosomal and mitochondrial impairment.

Finally, we investigated whether lysosomal damage affects lysosomal acidification in tNeurons. Compared with aged tNeurons, aged/sAD tNeurons exhibited a slight loss of lysosomal acidification at baseline, which was exacerbated by LLOME treatment (Extended Data Fig. 6f). Given that previous studies found that APP derivatives preferentially accumulate in poorly acidified lysosomes^43^, we examined whether Aβ42 deposits are proximal to lysosomes in aged/sAD tNeurons. Both APP-CTF and Aβ42 co-localized with LAMP1 (Fig. 3g and Extended Data Fig. 6g) or LC3B (Extended Data Fig. 6h) in aged/sAD tNeurons, providing support for a link between lysosomal damage and amyloid accumulation.

### Ageing and genetic link to organellar and calcium defects

**Extended Data Fig. 7:**
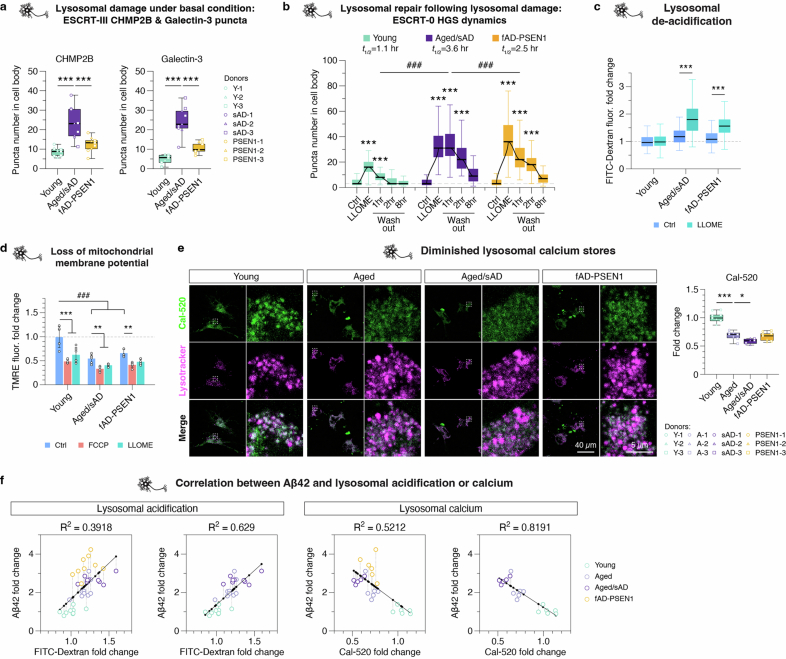
Abnormal lysosomal damage response links to defective organelle and calcium homeostasis in aged/sAD and fAD-PSEN1 tNeurons. (**a**) Quantification of numbers of ESCRT-III CHMP2B and Galectin-3 puncta in the cell body of young, aged/sAD and fAD-PSEN1 tNeurons in the absence of lysosomal damage insults. tNeurons: CHMP2B: *n* = 117 (young), 97 (aged/sAD) and 104 (fAD-PSEN1) cells; Galectin-3: *n* = 111 (young), 95 (aged/sAD) and 104 (fAD-PSEN1) cells. (**b**) Quantification of numbers of ESCRT-0 HGS puncta in the cell body of young, aged/sAD and fAD-PSEN1 tNeurons during lysosomal damage insults mediated by LLOME treatment, and recovery from lysosomal damage after LLOME washout for up to 8 hr. The half-life (*t*_*1/2*_) represents the time required for lysosomal repair. Young: *n* = 108 (Ctrl), 107 (LLOME), 111 (Washout 1 hr), 118 (Washout 2 hr) and 90 (Washout 8 hr) cells; Aged/sAD: *n* = 115 (Ctrl), 115 (LLOME), 117 (Washout 1 hr), 109 (Washout 2 hr) and 79 (Washout 8 hr) cells; fAD-PSEN1: *n* = 117 (Ctrl), 117 (LLOME), 113 (Washout 1 hr), 116 (Washout 2 hr) and 85 (Washout 8 hr) cells. (**c**) Changes in lysosomal acidification revealed by the intensity of preloaded pH-sensitive FITC-conjugated Dextran in young, aged/sAD and fAD-PSEN1 tNeurons at basal conditions or during 0.25 mM LLOME treatment for 30 min. Ctrl: *n* = 145 (young), 148 (aged/sAD) and 137 (fAD-PSEN1) cells; LLOME: *n* = 142 (young), 155 (aged/sAD) and 147 (fAD-PSEN1) cells. (**d**) Quantification of changes in mitochondrial membrane potential in young, aged/sAD and fAD-PSEN1 tNeurons at basal conditions and after the treatment of DMSO Ctrl, 20 µM FCCP or 0.25 mM LLOME for 30 min by analysing TMRE intensity. The values are revealed by a fold change relative to young tNeurons treated with DMSO. *n* = 6 (young), 6 (aged/sAD) and 6 (fAD-PSEN1) independent replicates. (**e**) Live-cell imaging and analysis of lysosomal calcium revealed by Cal-520, a fluorogenic calcium-sensitive indicator, conjugated with Dextran molecules, in young, aged, aged/sAD and fAD-PSEN1 tNeurons at basal conditions. Lysosomes are labelled with Lysotracker Red DND-99 (scale bar: 40 μm). *n* = 163 (young), 156 (aged), 187 (aged/sAD) and 139 (fAD-PSEN1) cells. Insert: higher magnification view of Cal-520 and Lysotracker Red DND-99 (scale bar: 5 μm). (**f**) Analysis of correlation between intracellular Aβ42 levels and lysosomal acidification or calcium in young, aged, aged/sAD and fAD-PSEN1 tNeurons. *n* = 9 independent replicates from three donors (lysosomal acidification) and *n* = 6 independent replicates from two donors (lysosomal calcium) from three independent experiments. Black solid line represents the fitted linear correlation. Coefficient of Discrimination (*R*^2^) is calculated using Pearson’s correlation. In panel **a**, **b**, **c**, and **e**, the boxes show median and 1^st^ and 3^rd^ quartile and the whiskers extending 1.5 times the interquartile range from the boxes. In panel **d**, data are displayed as mean ± SD. Data show mean and SD or box-and-whisker plots of two to three independent experiments and three cell lines from independent HC and AD donors. Statistical analysis is performed using One-Way ANOVA (**a**, and **e**) or Two-Way ANOVA (**b**, **c** and **d**) followed by Bonferroni post-hoc analysis. *P < 0.05, **P < 0.01 and ***P < 0.001. ###P < 0.001. Source numerical data are provided. Source data

We next investigated organelle homeostasis in fAD-PSEN1 tNeurons. Unlike aged/sAD tNeurons, fAD-PSEN1 tNeurons contained relatively lower-density accumulation of CHMP2B and galectin-3 puncta under basal conditions (Extended Data Fig. 7a). However, analysis of LLOME pulse-chase lysosomal repair kinetics revealed some similarities between aged/sAD and fAD-PSEN1 tNeurons, as both classes formed higher numbers of HGS puncta than young tNeurons during LLOME treatment (Extended Data Fig. 7b). Following LLOME washout, the repair kinetics of both AD tNeuron classes were slow relative to young tNeurons, the *t*_1/2_ of HGS puncta dissipation increased by approximately twofold in fAD-PSEN1 and threefold in aged/sAD tNeurons.

Analysis of lysosomal acidification revealed similar phenotypic parallels between fAD-PSEN1 and aged/sAD tNeurons (Extended Data Fig. 7c). The mitochondrial membrane potential was reduced in both aged/sAD and fAD-PSEN1 tNeurons at basal conditions and declined further after FCCP or LLOME treatment (Extended Data Fig. 7d). Although ageing seems to be a primary driver of constitutive lysosomal damage, both sAD and fAD-PSEN1 exhibit impaired LQC and reduced mitochondrial metabolism.

Given that previous studies revealed lysosomal calcium dysregulation underlying the pathological process in AD^44,45^, we also measured lysosomal calcium stores in tNeurons using Cal-520 (a fluorogenic calcium-sensitive indicator) conjugated to Dextran molecules. We found lysosomal calcium stores to be much lower in aged tNeurons and even less in aged/sAD and fAD-PSEN1 tNeurons at basal conditions compared with young tNeurons (Extended Data Fig. 7e). As lysosomal acidification and calcium dysregulation are associated with neurons affected by AD, we calculated the correlation between intracellular Aβ42 and either lysosomal acidification or lysosomal calcium in young, aged and aged/sAD tNeurons. There was a much stronger correlation between Aβ42 deposits and lysosomal calcium dysfunction than with impaired lysosomal acidification (Extended Data Fig. 7f). Thus, decreased lysosomal calcium stores in tNeurons accompanies the increased vulnerability of the lysosome system in aged and AD tNeurons as well as the increased Aβ42 burden. Nonetheless, fAD-PSEN1 tNeurons had a much higher Aβ42 burden than expected from either lysosomal acidification and calcium deficits from the above correlation. These experiments suggest a nuanced synergy between ageing and AD to disrupt organelle homeostasis and lead to cell-autonomous proteotoxic Aβ42 deposits.

### Correlation between lysosomal damage and Aβ deposits in tNeurons

**Extended Data Fig. 8:**
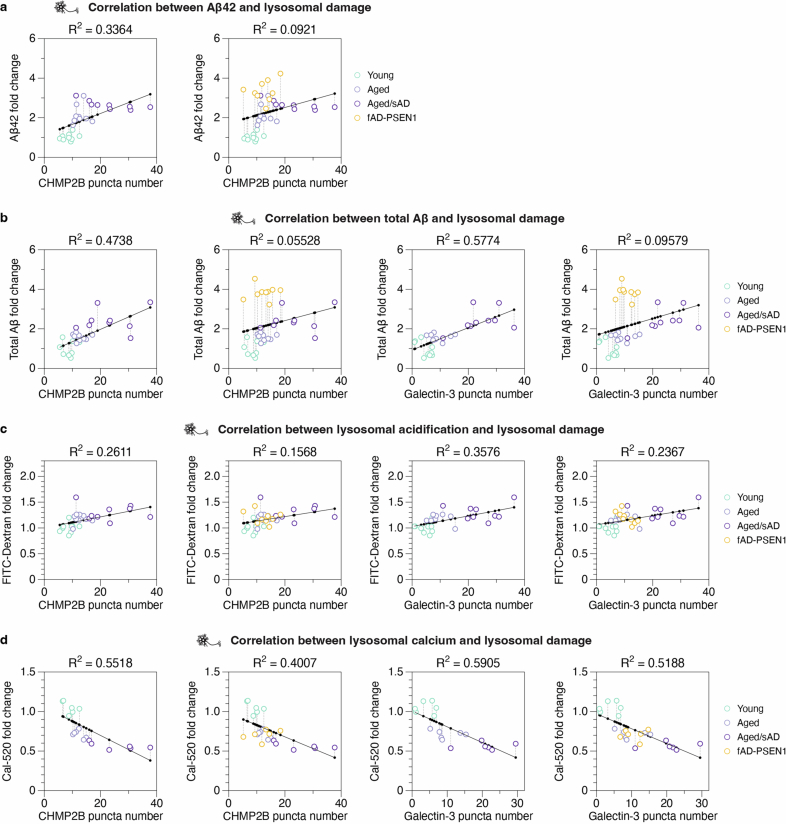
Lysosomal damage correlates with aging and intrinsic disease properties in AD patient neurons. (**a**) Analysis of correlation between intracellular Aβ42 levels and CHMP2B puncta numbers in young, aged, aged/sAD and fAD-PSEN1 tNeurons. (**b**) Analysis of correlation between intracellular total Aβ levels and Galectin-3 or CHMP2B puncta numbers in young, aged, aged/sAD and fAD-PSEN1 tNeurons. (**c**) Analysis of correlation between lysosomal acidification and Galectin-3 or CHMP2B puncta numbers in young, aged, aged/sAD and fAD-PSEN1 tNeurons. (**d**) Analysis of correlation between lysosomal calcium and Galectin-3 or CHMP2B puncta numbers in young, aged, aged/sAD and fAD-PSEN1 tNeurons. Panel **a**, **b** and **c**: *n* = 9 independent replicates from three donors and three experiments; Panel **d**: *n* = 6 independent replicates from two donors and three experiments. Black solid line represents the fitted linear correlation. Coefficient of Discrimination (*R*^2^) is calculated using Pearson’s correlation. Source numerical data are provided. Source data

The causal relationship between lysosomal damage and Aβ deposition in AD remains an important and poorly understood question^10–12,43,46,47^. Our characterization of both constitutive lysosomal damage and Aβ burden in the same set of tNeurons offered the possibility to assess their correlation in aged and AD tNeurons. When comparing young, aged and aged/sAD tNeurons, we observed a moderate positive correlation between Aβ42 deposits and the number of galectin-3 (Pearson’s coefficient of discrimination (*R*^2^) = 0.52; Fig. 3h) or CHMP2B puncta (*R*^2^ = 0.34; Extended Data Fig. 8a). A similar positive correlation was also observed between these lysosomal damage markers with intracellular total Aβ (Extended Data Fig. 8b). As above, the PSEN1-mutant fAD-PSEN1 tNeurons were outliers as they contained a higher Aβ42 burden than expected from the correlation measured for aged and aged/sAD tNeurons.

We also examined the correlation between the lysosomal damage markers CHMP2B and galectin-3 and deficits in either lysosomal acidification or calcium stores. Comparisons of the fold change in pH-sensing FITC–dextran revealed a mild positive correlation with the number of both galectin-3 and CHMP2B puncta (Extended Data Fig. 8c). Notably, the fold changes in the calcium-sensing Cal-520 revealed a stronger negative correlation with the damage markers (Extended Data Fig. 8d). In all cases, the correlation was ageing- and sAD-dependent. These analyses indicate that lysosomal deficits in ageing and AD are linked to lysosomal damage.

### Lysosomal damage pathology in the brains of mice and humans with AD

**Fig. 4:**
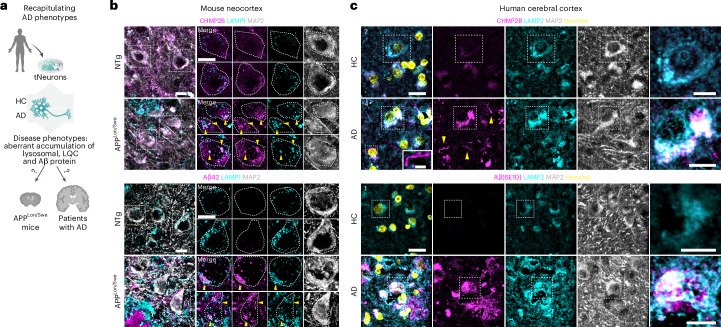
Lysosomal damage is linked to amyloid accumulation in post-mortem brain tissue. **a**, Schematic of the experimental pipeline to test whether the disease phenotypes observed in AD tNeurons are also detected in brain tissue of patients with AD and transgenic mice expressing mutant human APP with the Swedish and London mutations (APP^Lon/Swe^) for modelling AD. **b**, Immunofluorescence staining of CHMP2B, Aβ42 and LAMP1 in the neocortex of non-transgenic mice (NTg) and APP^Lon/Swe^ transgenic mice (left). The brain tissue was co-stained with MAP2. Higher magnification view of the regions in the dotted white boxes showing co-localization of CHMP2B, Aβ42 and LAMP1 in individual neurons are provided (right). The yellow arrowheads point to intraneuronal co-localization of CHPM2B and Aβ42 with LAMP1. Scale bars, 10 μm. **c**, Immunofluorescence staining of CHMP2B, Aβ(6E10) and LAMP2 in the cerebral cortex of human donors (HC and AD). The brain tissue was co-stained with MAP2 and Hoechst. Higher magnification views of the regions in the white boxes showing CHMP2B and Aβ(6E10) co-localization with LAMP2 are provided (right). Numbers (1, 2 and 4) indicate the case number associated with the representative images. Scale bars, 20 μm (main images), 5 μm (inset in image 4, left) and 10 μm (magnified views, right). The yellow arrowheads point to CHMP2B^+^ fibril structures.

**Extended Data Fig. 9:**
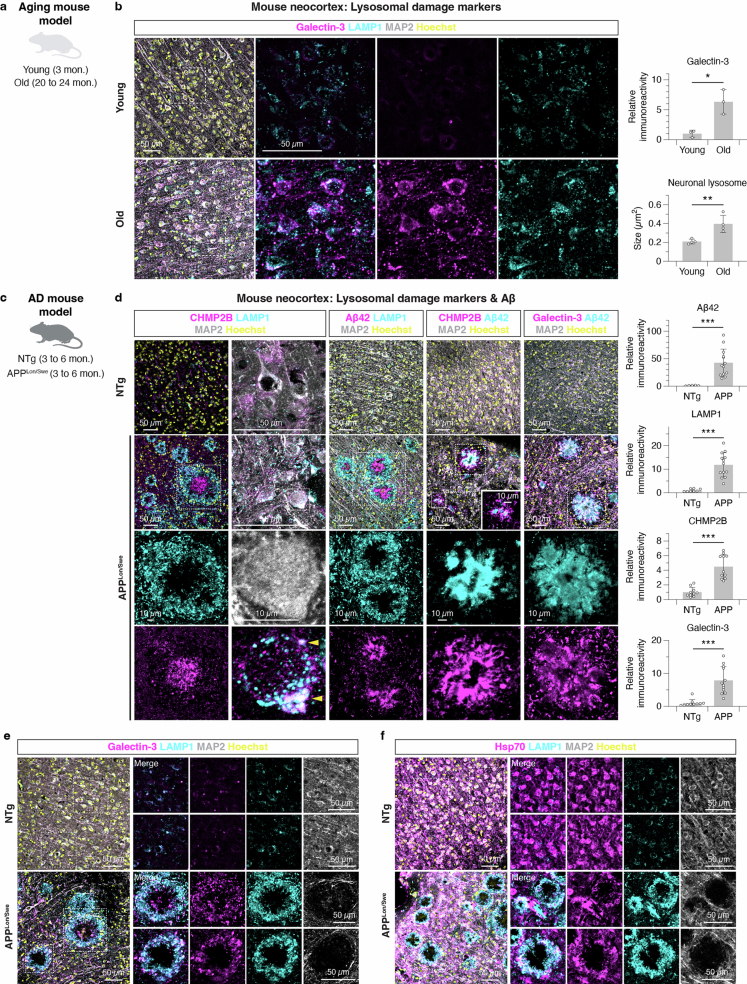
Lysosomal damage markers are elevated in the cortex of old mice and associated with neuropathological signatures of AD in the cortex of APP transgenic mice. (**a**) Experimental schematic for wild-type young (3-month old) and old (20 to 24-month old) mice. (**b**) Representative images and IF quantification of Galectin-3 immunoreactivity and lysosomal size based on LAMP1 signals in neurons of wild-type young and old mice. Galectin-3: *n* = 3 mice; Lysosomal size: *n* = 4 mice; Scale bar: 50 μm. (**c**) Experimental schematic for non-transgenic wild-type mice (NTg) and transgenic mice expressing mutant human APP with the Swedish (K670N/M671L) and London (V717I) mutations (APP^Lon/Swe^) (3 to 6-month old). (**d**) IF staining and quantification of Aβ42, LAMP1, CHMP2B and Galectin-3 in the neocortex of NTg and APP^Lon/Swe^ transgenic mice, related to Fig. 4b. Extracellular and intraneuronal co-localization of CHPM2B with LAMP1 in the brain of APP^Lon/Swe^ mice. The brain tissue is co-stained with MAP2 and Hoechst (scale bar: 50 μm). Aβ42: *n* = 8 (NTg) and 13 (APP^Lon/Swe^) mice; LAMP1: *n* = 8 (NTg) and 13 (APP^Lon/Swe^) mice; CHMP2B: *n* = 10 (NTg) and 11 (APP^Lon/Swe^) mice; Galectin-3: *n* = 10 (NTg) and 12 (APP^Lon/Swe^) mice. Insert: higher magnification view of co-localization between CHMP2B, Galectin-3, LAMP1 and Aβ42 (scale bar: 10 μm). Arrowhead: CHMP2B and LAMP1 co-localization in an individual neuron. (**e**) Immunostaining of Galectin-3 (magenta), LAMP1 (cyan) and MAP2 (grey) counterstained with Hoechst (yellow) in the cortex of NTg and APP^Lon/Swe^ mice (scale bar: 50 μm). Inset: higher magnification view of LAMP1-positive inclusions (scale bar: 50 μm). (**f**) Immunostaining of Hsp70 (magenta), LAMP1 (cyan) and MAP2 (grey) counterstained with Hoechst (yellow) in the cortex of NTg and APP^Lon/Swe^ mice (scale bar: 50 μm). Inset: higher magnification view of LAMP1-positive inclusions (scale bar: 50 μm). Panel **b** and **d** are displayed as mean ± SD from three to four independent experiments in aging and AD mice model. Statistical analysis is performed using two-sided Student’s *t*-test. *P < 0.05, **P < 0.01 and ***P < 0.001. Source numerical data are provided. Source data

To link our in vitro findings to in vivo pathophysiology, we carried out histopathological analyses of the post-mortem brain cortex of patients with AD and mouse models of ageing and AD (Fig. 4a). Compared with young mice (3 months), aged mice (20–24 months) exhibited a moderate increase in galectin-3 immunoreactivity, which co-localized with enlarged LAMP1^+^ lysosomes in neurons (Extended Data Fig. 9a,b). We also examined brains from an AD mouse model expressing human APP carrying the Swedish (K670N/M671L) and London (V717I) mutations (APP^Lon/Swe^)^48^. Similar to aged/sAD tNeurons, APP^Lon/Swe^ brains exhibited widespread accumulation of LAMP1^+^ clumps co-localizing with CHMP2B, galectin-3 and Hsp70, which were absent in non-transgenic mice brains (Fig. 4b and Extended Data Fig. 9c–f). These damaged lysosomal clumps were observed intracellularly, in the perinuclear region in individual pyramidal neurons as well as in the extracellular space devoid of MAP2 staining, which is suggestive of severe neuronal death linked to lysosomal damage. Notably, pyramidal neurons proximal to Aβ plaques contained intraneuronal Aβ42 deposits within lysosomes in APP^Lon/Swe^ mice (Fig. 4b).

**Extended Data Fig. 10:**
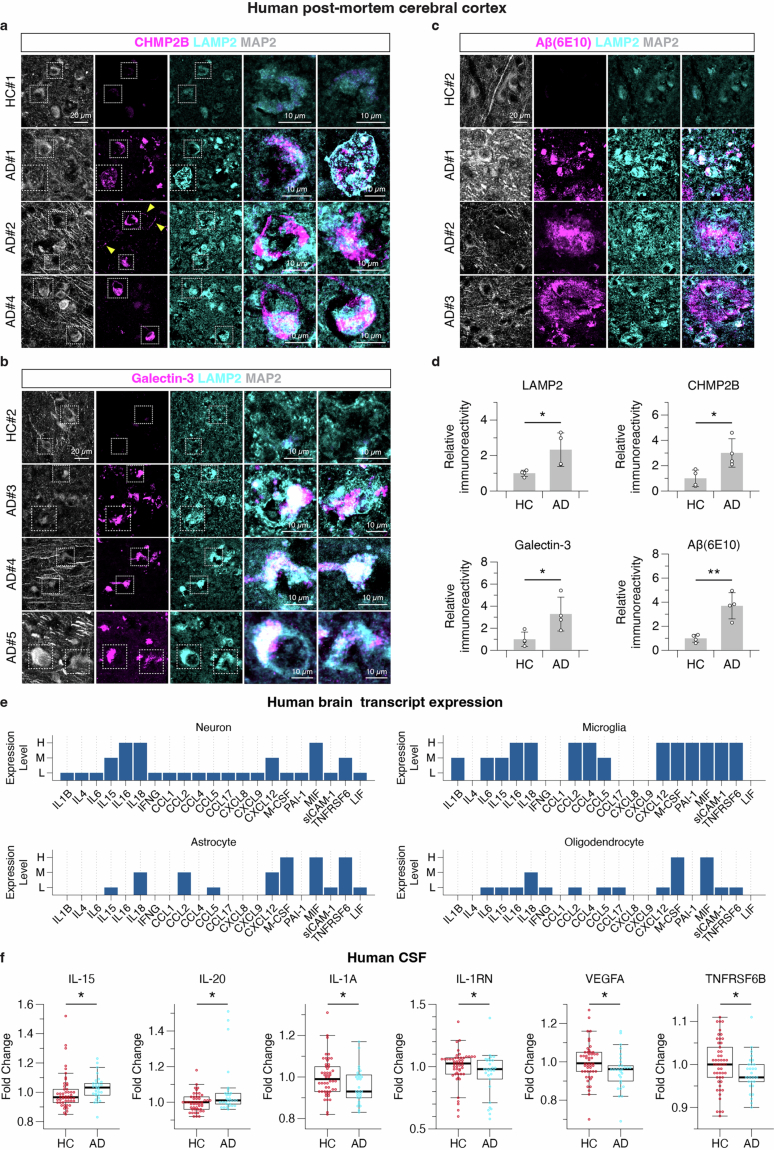
AD patients show prominent lysosomal damage phenotypes in brain tissue and elevated inflammatory factor secretion in CSF samples. (**a**–**c**) Immunostaining of LAMP2 (cyan) and MAP2 (grey) along with CHMP2B (**a**), Galectin-3 (**b**) or Aβ(6E10) (**c**) (magenta), in the post-mortem cerebral cortex of HC and AD donors, related to Fig. 4c (scale bar: 20 μm). #: indicates images acquired from different individuals. Inset: higher magnification view of LAMP2-positive inclusions within individual neuron (scale bar: 10 μm). (**d**) IF quantification of LAMP2, CHMP2B, Galectin-3 and Aβ(6E10) in the post-mortem cerebral cortex of HC and AD donors. HC: *n* = 4; AD: *n* = 4 individuals. (**e**) Secreted inflammatory factors by human tNeurons are detected in human brain cells. Single-cell transcriptomic analysis of selected cytokines and chemokines from Fig. 5d,e in major cell types of human brain, including neuron, astrocyte, microglia and oligodendrocyte. The transcript expression scores (H: high; M: medium; L: low) is determined by the expression level of each transcript in public datasets retrieved from the Human Cell Atlas and Allen Brain Map. (**f**) Quantification of changes in the levels of inflammatory panel biomarkers measured in human CSF from HC and AD donors using the SOMAScan assay platform. HC: *n* = 50; AD: *n* = 29 individuals. In panel **d**, IF data are displayed as mean ± SD. In panel **f**, the boxes show median and 1^st^ and 3^rd^ quartile and the whiskers extending 1.5 times the interquartile range from the boxes. Statistical analysis is performed using two-sided Student’s *t*-test. *P < 0.05, **P < 0.01. Source numerical data are provided. Source data

Brain tissue of patients with AD also exhibited intraneuronal and global elevation of Aβ, CHMP2B and galectin-3 immunoreactivity co-localized with LAMP2 compared with healthy control (HC) individuals (Fig. 4c and Extended Data Fig. 10a–d). Similar to our findings in tNeurons, we observed increased co-localization of CHMP2B and galectin-3 with LAMP2 in the brains of individuals with AD. Interestingly, we identified two distinct patterns of CHMP2B staining in the brains of patients with AD. CHMP2B mainly co-localized with LAMP2^+^ lysosomes in the perinuclear region of neurons but also formed thin thread-like structures of variable length devoid of LAMP2 staining. Using anti-Aβ (6E10), we observed intraneuronal and extracellular Aβ/APP deposits associated with LAMP2^+^ lysosomes in the brains of patients with AD (Fig. 4c and Extended Data Fig. 10c). Despite their complexity, the brains of patients with AD and mice models indicate an intraneuronal and global increase in lysosomal damage accompanying co-aggregation with amyloid plaques and neuritic degeneration that are consistent with the phenotypes of our tNeuron models.

### Cell-autonomous inflammatory activation in AD tNeurons

**Fig. 5:**
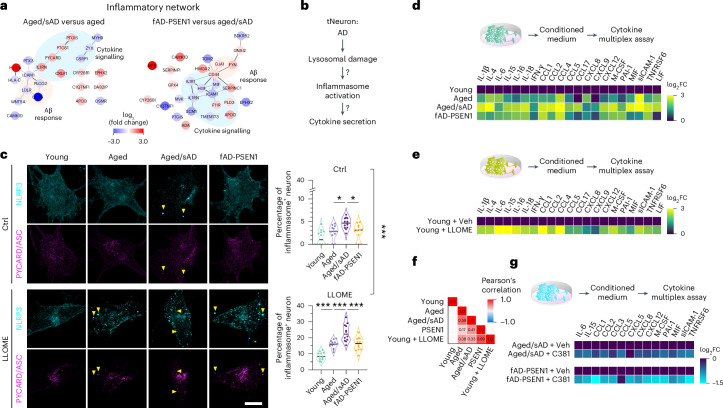
Lysosomal damage mediates inflammatory responses in AD tNeurons. **a**, Interaction network of proteins involved in the inflammatory response pathway in tNeurons. The relative abundance is shown as the log_2_-transformed fold change (log_2_FC); increase in red and decrease in blue. **b**, Schematic of the test to determine whether lysosomal damage is linked to inflammasome activation and cytokine secretion in AD neurons. **c**, Inflammasome activation. Representative images of tNeurons immunostained for the inflammasome markers NLRP3 and PYCARD/ASC following treatment with or without 0.25 mM LLOME for 3 h on PID 40 (left). The yellow arrowheads point to co-localization of NLRP3 and PYCARD/ASC. Scale bar, 10 μm. Percentage of tNeurons showing inflammasomes in each image (right). The median of the data is shown; *n* = 441–477 young, 399–513 aged and 522–648 aged/sAD cells from four donors and three independent experiments. **d**, Inflammatory profiling of the conditioned medium from all groups of tNeurons on PID 40 under basal conditions. The cytokine and chemokine log_2_FC values are relative to those for young tNeurons; *n* = 6 young, 6 aged, 12 aged/sAD and 4 fAD-PSEN1 independent replicates from two experiments. **e**, Inflammatory profile of the conditioned medium from young tNeurons with or without chronic lysosomal damage stress (0.1 mM LLOME for 7 d starting at PID 33). The cytokine and chemokine log_2_FC values are relative to the vehicle control (DMSO); *n* = 6 young + vehicle and 8 young + LLOME independent replicates from two experiments. **f**, Pearson’s correlation analysis of the identified cytokines and chemokines. The Pearson’s correlation coefficients are indicated on the graph. **g**, Inflammatory profiling of the conditioned medium from aged/sAD and fAD-PSEN1 tNeurons at PID 35 following treatment with or without 3.1 µM C381 for 7 d. The log_2_-transformed FC in mean fluorescence intensity relative to the vehicle control (DMSO) was determined; *n* = 4 independent replicates (all groups) from two experiments. **c**–**e**,**g**, Statistical analysis was performed using a two-sided Student’s *t*-test (**g**), or one-way (**d**,**e**) or two-way (**c**) ANOVA, followed by Bonferroni’s post-hoc analysis. *P < 0.05 and ***P < 0.001. Veh, vehicle. Source numerical data are provided. Source data

Our proteomic analysis showed that aged/sAD tNeurons upregulate a protein network involved in cytokine signalling, including the inflammasome adaptor protein PYCARD/ASC (Fig. 5a). Activation of NLRP3 inflammasomes in microglia has been extensively linked to AD^49^, we thus investigated whether this inflammatory response is also elevated in AD neurons and associated with lysosomal damage (Fig. 5b). Immunofluorescence analyses showed that, under basal conditions, aged/sAD donors contained slightly more inflammasome-positive neurons than aged and fAD-PSEN1 donors (Fig. 5c). Following a 3-h LLOME treatment, the inflammasome-positive neurons were increased by 35–40% in aged/sAD tNeurons compared with 18–30% in aged and fAD-PSEN1 tNeurons. We conclude that AD neurons have cell-autonomous inflammasome activation, potentially associated with their higher vulnerability to lysosomal damage.

We next examined whether AD tNeurons secrete inflammatory factors. Multiplex cytokine profiling, using Luminex assays, of the conditioned medium from untreated tNeurons showed that aged/sAD tNeurons have increased secretion of pro-inflammatory (for example, interleukin (IL)-1β) and anti-inflammatory (for example, IL-15) cytokines and chemokines (for example, chemokine ligand 2 (CCL2); Fig. 5d and Supplementary Fig. 1). To assess whether lysosomal damage indeed promotes secretion of inflammatory factors, we subjected young tNeurons to low-dose LLOME treatment to elicit a chronic, sublethal lysosomal damage state. This treatment led to increased secretion of IL-1β, IL-6, interferon (IFN)-γ and CCL2 (Fig. 5e and Supplementary Fig. 1). Using Pearson’s correlation analysis, we identified a moderate and positive correlation between cytokine secretion, ageing, AD and lysosomal damage (Fig. 5f). This led us to hypothesize that rescue of lysosomal deficits should reduce the secretion of inflammatory factors. We thus treated tNeurons with C381, a lysosome-targeting small molecule that promotes lysosomal acidification and resilience to damage^50^. Strikingly, the amelioration of lysosomal damage by C381 in aged/sAD and fAD-PSEN1 tNeurons reduced the secretion of IL-6, IL-15 and CCL2 (Fig. 5g and Supplementary Fig. 2). These experiments link ageing- and AD-dependent lysosomal damage to neuron-autonomous secretion of inflammatory cytokines.

To relate these cell-based findings to a physiological disease-relevant context, we examined cytokine expression in published human datasets of single-nucleus transcriptomic analyses of post-mortem cortex from HC donors (Extended Data Fig. 10e). Neurons were confirmed to express cytokine and chemokine transcripts, albeit at lower levels than microglia, which have high transcript levels given their immune-active function^28^. We also conducted an unbiased analysis of cerebrospinal fluid (CSF) proteins in 50 HC and 29 AD donors in a search for biomarkers (Supplementary Table 3). Notably, both the conditioned medium from aged/sAD tNeurons and CSF from AD patients were found to have elevated IL-15 (Fig. 5d and Extended Data Fig. 10f), which has been positively correlated with age of AD onset^51^.

### Rescue of lysosomal function ameliorates AD pathologies

**Fig. 6:**
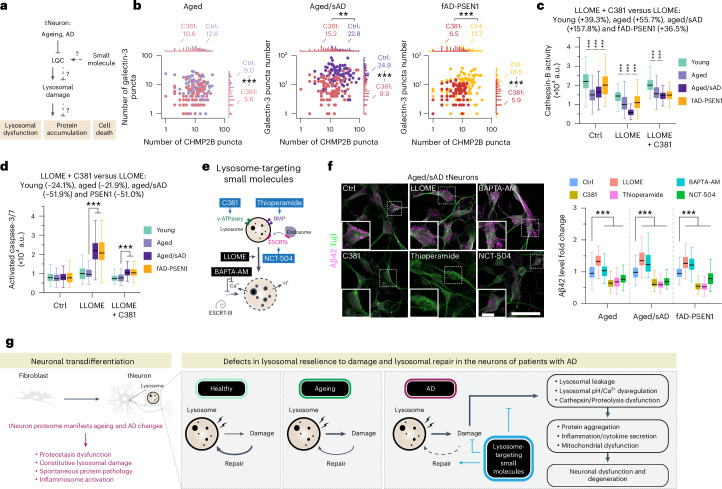
Pharmacological improvement of lysosomal resilience to damage ameliorates AD phenotypes in tNeurons. **a**, Schematic of tests to determined whether LQC rescue provides neuroprotective effects in AD tNeurons. **b**, Concurrent change in the number of CHMP2B and galectin-3 puncta by treatment with 0.25 mM LLOME for 30 min at PID 35 following pretreatment with DMSO (Ctrl) or 3.1 µM C381 for 7 d. Ctrl, *n* = 103 aged, 95 aged/sAD and 104 fAD-PSEN1 cells; C381, *n* = 88 aged, 95 aged/sAD and 91 fAD-PSEN1 cells. Each dot represents the number of detectable CHMP2B and galectin-3 puncta in an individual neuron. **c**, Changes in cathepsin-B activity caused by 0.25 mM LLOME treatment for 30 min at PID 35 following pretreatment with 3.1 µM C381 for 7 d. Young, *n* = 145 Ctrl, 142 LLOME and 147 LLOME + C381 cells; aged, *n* = 157 Ctrl, 156 LLOME and 122 LLOME + C381 cells; aged/sAD, *n* = 157 Ctrl, 152 LLOME and 155 LLOME + C381 cells; fAD-PSEN1, *n* = 142 Ctrl, 142 LLOME and 141 LLOME + C381 cells. **d**, Levels of caspase-3/7 activation after 0.5 mM LLOME treatment for 1 h at PID 42 following pretreatment with 3.1 µM C381 at PID 35 for 7 d. Young, *n* = 162 Ctrl, 142 LLOME and 105 LLOME + C381 cells; aged, *n* = 158 Ctrl, 146 LLOME and 133 LLOME + C381 cells; aged/sAD, *n* = 148 Ctrl, 167 LLOME and 142 LLOME + C381 cells; fAD-PSEN1, *n* = 136 Ctrl, 147 LLOME and 148 LLOME + C381 cells. **e**, Schematic showing small molecules that modulate lysosomal function and damage. Small molecules with beneficial effects are labelled in blue and those with detrimental effects are labelled in black. **f**, Effects of small molecules (treatment with 0.25 mM LLOME, 2.5 µM BAPTA-AM, 3.1 µM C381, 5 µM thioperamide or 2.5 µM NCT-504 for 2 d) on intraneuronal Aβ42 levels in aged, aged/sAD and fAD-PSEN1 tNeurons. Representative images of aged/sAD tNeurons immunostained for Aβ42 and Tuj1 at PID 35 (left). Insets: higher magnification views of the regions in the dashed boxes showing Aβ42 in individual neurons. Scale bars, 50 μm (main images) and 10 μm (insets). Fold changes in Aβ42 levels, determined from the immunofluorescence images, during small-molecule treatment relative to the DMSO Ctrl; *n* = 104–122 (aged), 88 to 144 (aged/sAD) and 61–136 (fAD-PSEN1) cells. **b**–**d**,**f**, Data are from three donors and three independent experiments. **g**, We propose that in AD—either as a result of stochastic events or mutational burdens—lysosomal repair defects are exacerbated, leading to overwhelmed LQC machineries and the sustained presence of damaged lysosomes. Restoration of lysosomal homeostasis and damage ameliorate AD pathologies in neurons. **c**,**d**,**f**, The boxes show the median and first and third quartiles (box boundaries), and the whiskers extend 1.5× the interquartile range from the boxes. **b**–**d**,**f**, Statistical analysis was performed using a two-sided Student’s *t*-test (**b**) or a one-way ANOVA (**c**,**d**,**f**), followed by Bonferroni’s post-hoc analysis. **P < 0.01 and ***P < 0.001. Source numerical data are provided. Source data

We next used C381 to further investigate the link between lysosomal impairment, lysosomal dysfunction and neuronal cell death in AD tNeurons (Fig. 6a). Pre-incubation of aged and AD tNeurons with C381 concurrently reduced the number of constitutive CHMP2B and galectin-3 puncta on lysosomes (Fig. 6b), providing support for its ability to rescue lysosomal deficits. We next measured how C381 treatment of tNeurons affects additional lysosomal functions. Given that lysosomal hydrolases, such as cathepsin-B, are optimally active at pH 4–5, lysosomal de-acidification caused by ageing, AD or LLOME should abrogate their activity. Cathepsin-B hydrolysis of the fluorogenic substrate Magic Red was indeed reduced in aged and aged/sAD tNeurons under basal conditions and was dramatically lost following LLOME treatment (Fig. 6c). However, C381 treatment restored cathepsin-B activities in LLOME-treated AD tNeurons to near-normal levels. The lysosomal vulnerability of AD tNeurons rendered them exquisitely sensitive to LLOME-mediated cell death, as measured by caspase-3/7 activation (Fig. 6d). This phenotype was abrogated by C381 rescue of lysosomal function, which was strongly neuroprotective from lysosome-mediated apoptosis in AD tNeurons.

The availability of small molecules that can either improve or further impair lysosomal homeostasis was next exploited to test whether lysosomal dysfunction contributes to cell-autonomous Aβ42 deposits in aged and AD tNeurons (Fig. 6e,f). To increase lysosomal damage, we used LLOME or the calcium chelator BAPTA-AM. These treatments increased intraneuronal Aβ42 levels in both aged/sAD and fAD-PSEN1 tNeurons. We used three mechanistically distinct compounds to improve lysosomal function. In addition to C381, we used thioperamide, which increases lysosomal phospholipid BMP^52^ and NCT-504, which upregulates ESCRT transcripts^53^. Strikingly, incubating AD tNeurons with either of these compounds reduced the levels of Aβ42 deposits in aged and AD tNeurons by about 20–46% (Fig. 6f). These experiments support a causal connection between neuronal lysosomal dysfunction and cell-intrinsic generation of intraneuronal Aβ42 deposits. They further demonstrate that ameliorating lysosomal function can by itself reduce Aβ42 burdens in AD neurons.

## Discussion

Here we demonstrate that neuronal transdifferentiation provides a powerful approach to study cellular and mechanistic aspects of human brain ageing and AD. Although induced pluripotent stem cells have great potential to model human genetic disorders^54^, the cellular rejuvenation process erases access to the contribution of ageing to neurodegenerative diseases^15,16,55,56^. Building on previous studies showing that human tNeurons preserve hallmarks of ageing^16,56–58^, we harnessed tNeurons to reveal proteomic signatures and identify proteostasis and LQC pathways as selectively affected by ageing and AD (Fig. 6g). Our proteomic analyses resonate with the genome-wide and proteome-wide association studies identifying AD causal and risk genes, such as *CLU*, *PLD3* and *SNX32*, belonging to the endosome–lysosomal pathway^8,59^. We propose that LQC defects are an early pathogenic event that causes neurons to experience sustained stress and deterioration, leading to cell-autonomous formation of proteotoxic deposits, mitochondrial dysfunction, inflammasome activation and risk of neurodegeneration. This may suggest that ageing and AD-linked mutations act in a two-hit process. In the first hit, neurons enter a vulnerable phase with impaired proteostasis and lysosomal homeostasis. Persistent lysosomal damage and LQC defects diminish neuronal resilience to counteract harmful insults, such as mutations or sporadic stressful events, that would constitute the second hit, triggering the degenerative process. In turn, these defects may then elicit cell non-autonomous mechanisms, such as inflammation or spreading of toxic aggregates, that aggravate overt neuronal loss across brain regions. Our work further suggests that tNeurons could be a reliable tool for evaluating small molecules possessing neuroprotective effects against AD pathologies.

Age-dependent impairment of lysosomal function has been observed along a longitudinal ageing axis in different model systems^3,5,60^. Lysosomal function is also impaired in AD and brain tissues from patients with AD are extensively characterized by the co-occurrence of Aβ and lysosomal pathology including amyloid plaques enriched in lysosomal proteins^61–63^. The causal relationship between amyloid accumulation and lysosomal damage is still poorly understood. There is a long-standing hypothesis that increased endocytosis of extracellular protein oligomers causes lysosomal damage, leading to seeding of protein aggregates and ultimately neuronal death in AD^10–12^. Recent studies reported that intrinsically perforated endosome–lysosomes are present in diseased neurons and facilitate the seeding of cytoplasmic aggregates following internalization of preformed fibrils^64,65^. Our tNeuron models provide valuable insights into this important problem. Here we show that constitutive disruptions in lysosomal membrane integrity and reduced lysosomal repair mechanisms are increased by ageing and more by late-onset sAD. These phenotypes are correlated with, but may not solely depend on, intracellular Aβ. One possibility is that the tNeuron phenotypes reflect a very early stage of dysfunction in AD. Although we did not seed with fibrils in this study, it is possible that lysosomal dysfunction leads to elevated Aβ levels that are then secreted from AD tNeurons; subsequent uptake of extracellular Aβ would further aggravate lysosomal damage phenotypes. The vicious cycle proposed by our model would eventually lead to severe collapse of endosome–lysosomal homeostasis essential for neuronal survival. Importantly, our experiments also suggest this vicious cycle might be interrupted by small molecules that ameliorate lysosomal function.

The measurement of in vivo lysosomal pH in transgenic mouse models for AD indicated that the emergence of lysosomal acidification deficits precedes Aβ depositions and that as disease progresses, neurons build up Aβ/APP-CTF selectively in perinuclear de-acidified lysosomes, leading to lysosomal damage^43^. However, our tNeuron models do not fully recapitulate this phenotype. We only observed a subtle deficit in lysosomal acidification in AD tNeurons at baseline, similar to previous findings in cells lacking *PSEN1*/*2* or *PLD3*—gene variants that are linked to early and late-onset AD, respectively^66,67^—but we detected a greater lysosomal acidification deficit under stressful conditions. This could be because tNeurons detect early manifestations in AD pathology and investigating early triggers of endosome–lysosomal dysfunctions present substantial challenges in transgenic AD mice or human brains. The LQC machineries are likely to be severely impaired by the time of analysis, rendering them incapable of restoring lysosomes, resulting in lysosomal de-acidification. Interestingly, we observed lysosomal calcium dysregulation in aged and AD tNeurons under basal conditions and found it correlated better with lysosomal damage and intracellular Aβ levels. This raises an important point remaining to be determined concerning the relative contribution of lysosomal acidification or calcium deficits as early triggers of AD^67,68^. Increasing attention is being paid to intracellular accumulation of amyloids in AD associated with intrinsic lysosomal defects during the early stages of disease^43,46^. Mass cytometry analysis of human post-mortem brain demonstrated that neurons accumulating intracellular Aβ are preferentially lost early during the progression of AD in contrast to tau, which preferentially accumulates in neuronal subtypes resilient to neuronal loss^46^. The accumulation of Aβ/APP-CTF promotes neuropathy by eliciting endosomal abnormality and Rab5 overactivation, and compromising lysosomal calcium stores through inhibition of lysosome–endoplasmic reticulum contacts in *PSEN1*-knockout or mutant mouse neurons, or human induced neurons^45,69–71^. Interestingly, we found that the severity of lysosomal phenotypes in fAD-PSEN1 tNeurons much resemble those of aged tNeurons, suggesting that this genetic variant and ageing make comparable contributions to neuronal lysosomal defects, at least under basal conditions. When exposed to additional insults, aged/sAD and fAD-PSEN1 tNeurons are undoubtfully highly vulnerable to lysosomal stress. As PSEN1 is directly involved in APP processing and Aβ generation, it is possible that in these cells Aβ accumulation precedes lysosomal dysfunction, and these deposits eventually overwhelm LQC and lead to constitutive lysosome damage.

Damage accumulation is an inevitable feature of ageing in living organisms. To ensure cellular homeostasis, it is essential to repair and remove damaged proteins and/or organelles to slow ageing. Growing evidence indicates that lysosomal clearance declines with age and age-related diseases^72,73^. However, it is yet to be understood how LQC pathways are affected by ageing and human disease. Our results uncover an age-dependent decline in ESCRT- and galectin-mediated LQC that promotes cell-autonomous proteostasis deficits, cytokine secretion and cell death in tNeurons of patients with AD. Notably, recent studies suggested that molecular chaperones, PI4K2A, ORP family members, LRRK2, annexin A7 and stress granules are also involved in LQC^32,33,74–77^. Due to the increasing complexity of the LQC machinery, it would be of interest to evaluate other LQC pathways to understand their impact on ageing and AD in the future. Surprisingly, our findings that CHMP2B, LAMP1, Hsp70, Aβ and TDP-43 become associated with damaged lysosomes in neurons are reminiscent of phenotypes linked to granulovacuolar degeneration (GVD). GVD is a typical AD hallmark, which is characterized by the presence of cytoplasmic granule-containing vacuoles, termed GVD bodies^78^. Revealed by neuropathological observations in post-mortem brain tissue of AD patients, GVD bodies are selective to pyramidal neurons, structurally resemble endocytic and autophagic vesicles, and contain a variety of proteins, including the typical hallmark proteins, as aforementioned^78,79^. However, the pathological mechanisms of their formation remain mostly unclear. Our in vitro and in vivo findings may suggest that lysosomal damage could be involved in GVD aetiology during the development of AD. In addition, our tNeuron proteomics revealed a decrease in levels of PLD3 in ageing and sAD. PLD3 is a lysosomal exonuclease enriched in the senile plaques in the brain of patients with AD^66,80,81^. The levels of PLD3 transcripts and proteins are reduced in the brain of individuals with AD. Depletion of PLD3 leads to lysosomal dysfunction and lipid accumulation, and activation of the cGAS–STING signalling mediated by cytosolic nucleotides leaked out from lysosomes^66^. STING is a substrate of lysosomal degradation to attenuate the activation of cGAS–STING signalling and control pro-inflammatory cytokine expression^82^. However, STING is also a driver of autophagy activation. The failure in lysosomal degradation of STING aberrantly activates Atg5-dependent autophagy, which amplifies immune responses and a variety of cell-death pathways^83^. Previous findings also indicated that lysosomal membrane permeabilization in microglia activates NLRP3 inflammasomes and promotes the secretion of pro-inflammatory cytokines, thus aggravating neuronal damage^84^. Our study indicates that constitutive lysosomal damage promotes inflammasome activation in AD tNeurons, consistent with a recent study that indicates AD tNeurons recapitulate senescence-like neurons observed in the brain of patients with AD as a novel source of neuroinflammation^20^.

There are limitations to our study. We used monocultures of tNeurons without glial cell support and short-term culture conditions (that is, 5–6 weeks), which are quite distinct from the complex tissue organization and prolonged ageing processes in the human brain. Thus, our transdifferentiated cell-based models may not entirely reflect the physiological signatures of ageing and AD. The neurons are derived from human adult fibroblasts of several donors to account for patient heterogeneity. The challenging conversion and cell culture system limit the experimental scalability for larger donor cohort sizes and large-scale biochemical analyses. In addition, healthy young and aged fibroblasts were collected from donors who were clinically normal at collection but we cannot rule out their possibility of developing dementia and AD due to unidentified genetic variations or environmental factors in subsequent decades. Ageing affects many branches of proteostasis^5,85,86^, as demonstrated by our finding of increased p62, Ub^+^ and HspB1 with ageing and AD as well as increases in pathogenic protein deposits and impaired lysosomal and mitochondria function. Therefore, cellular health relies on the interplay among different proteostasis pathways, raising the question of which are the upstream triggers of ageing-associated proteostasis decline. How the entire LQC pathways are impaired by ageing and AD remains to be comprehensively investigated in future studies.

In summary, we propose that tNeurons provide insights into early neuron-intrinsic cell biological processes by which loss of proteostasis and organelle homeostasis contribute to AD pathogenesis. These insights would be impossible to obtain in intact brains, where the complex interplay between cell types in the tissue establishes a vicious cycle that probably exacerbates all responses. They would also not be possible in stem cell-derived neurons, which lack the ageing-linked phenotypes essential to these late-onset diseases, such as AD. One corollary of our experiments is that counteracting intrinsic proteostasis and lysosomal homeostasis deficits in aged and AD tNeurons may be attractive strategies for early stage prevention of the cascade of deleterious events in affected AD brains.

## Methods

Experiments involving cell culture studies were conducted according to a protocol reviewed and approved by Stanford University. All animal care and procedures complied with the Animal Welfare Act and were in accordance with institutional guidelines and approved by the V. A. Palo Alto Committee on Animal Research and the institutional administrative panel of laboratory animal care at Stanford University. Details of key resources used in this study are provided in a Key Resource Table available on *Zenodo* (10.5281/zenodo.14606908). Detailed protocols used in this study can be accessed on *protocols.io*^87–92^.

### Experimental model and participant details

#### Human participants

De-identified human fibroblasts, post-mortem prefrontal cortex and CSF samples from individuals of various ages and disease conditions were acquired from the Stanford Alzheimer’s Disease Research Center (ADRC). Cells and tissue samples were obtained from all participants under written consent approved by Institutional Review Board of Stanford University. The cell and tissue samples collected by the Stanford ADRC were not specifically for this study. For the histological experiments, among these participants, eight were assessed as HCs and ten were patients with cognitive impairment (dementia due to AD). For the CSF proteomics experiments, 50 participants were assessed as HCs and 29 were patients with cognitive impairment (mild cognitive impairment or dementia due to AD). Age and sex demographics are detailed in Supplementary Tables 1 and 3. Fibroblasts were collected at the Stanford ADRC and the Coriell Institute from cognitive normal young and aged donors as well as individuals with sAD or fAD showing the clinical symptoms of AD, including progressive cognitive impairment. At the Stanford ADRC individuals with cognitive decline received neurological examinations and cognitive tests to determine cognitive status and consensus diagnosis by a team of neuropathologists. The pathological diagnosis of post-mortem tissues was made by microscopy examination of multiple brain regions using the Amyloid, Braak neurofibrillary degeneration and CERAD neuritic plaque scores.

#### HEK293T cells

HEK293T cells are derived from human embryonic kidney and were acquired directly from the American Type Culture Collection (CRL-1573). HEK293T cells were grown in culture medium (DMEM supplemented with GlutaMAX, 10% fetal bovine serum (FBS), 1% penicillin–streptomycin, 1% HEPES and 1% sodium pyruvate; Thermo Fisher Scientific), sterilized using a 0.22 µm vacuum filter (Thermo Fisher Scientific), in a 37 °C incubator with 5% CO_2_. The HEK293T cells were used for lentiviral production by transfecting the lentiviral vector of interest mixed with packaging and envelope plasmid. The cells were passaged every three days using trypsin–EDTA (Thermo Fisher Scientific).

#### Human fibroblasts

Primary human adult fibroblasts derived from clinically healthy adults and individuals diagnosed with AD were collected from shared resources in the Stanford ADRC and the Coriell Institute for Medical Research, which operates the NIGMS, NIA, NINDS cell repository. A detailed protocol for the culture of primary human fibroblasts is available^87^. Briefly, the cells were grown in culture medium (DMEM supplemented with GlutaMAX, 10% FBS, 1% penicillin–streptomycin, 1% MEM non-essential amino acids solution, 1% sodium pyruvate and 0.1% β-mercaptoethanol; Thermo Fisher Scientific) sterilized using a 0.22 µm vacuum filter and maintained in a 37 °C incubator with 5% CO_2_. The subculture of proliferating fibroblasts used for regular experiments and neuronal transdifferentiation was typically within 3–7 passages. Some fibroblast lines obtained with slightly higher passage numbers (no more than 12 passages) were used for neuronal transdifferentiation. The cells were passaged every six or seven days using trypsin–EDTA.

#### Human tNeurons

tNeurons were converted from human fibroblasts with low passage numbers using a combination of transcription factors and small molecules. This method was derived from previously reported protocols^17,93^ with some modifications. A detailed protocol is available^89^. Briefly, the lentiviral expression of *Brn2*, *Ascl1*, *Myt1l* and *Ngn2* (also referred to as BAMN factors) in human fibroblasts initiated neuronal reprogramming. Transduced cells underwent puromycin and polysialylated-neural cell adhesion molecule (PSA-NCAM) selection and were cultured in reprogramming medium (DMEM/F12:Neurobasal (1:1) medium with 2% B-27, 1% N-2, 0.25% GlutaMAX and 1% penicillin–streptomycin; Thermo Fisher Scientific) for 15 days and then switched to maturation medium (BrainPhys neuronal medium (STEMCELL Technologies) with 2% B-27, 1% N-2, 0.25% GlutaMAX and 1% penicillin–streptomycin) for an additional 15–22 days. The culture media were supplemented with BDNF and NT-3 (neurotrophic factors; Peprotech), doxycycline (effector for the Tet-On system; Cayman), forskolin (cAMP activator; Sigma-Aldrich), SB 431542 (TGF-β/activin/nodal inhibitor; TOCRIS), dorsomorphin (BMP inhibitor; TOCRIS) and XAV939 (Wnt inhibitor; Stemgent; removal from maturation medium). Human tNeurons resemble cortical glutamatergic neurons and were used for evaluation of the reprogramming efficiency, phenotypic characterization and small-molecule treatments after five weeks in culture in a 37 °C incubator with 5% CO_2_. Half the culture medium was replaced with fresh medium every 2–4 days throughout the lifetime of the culture. Further details are provided in the ‘Direct generation of neurons from human fibroblasts’ section.

#### Mice

All of the mice used in this study had a C57BL/6 genetic background. Mice of old age (20–24-months old) were obtained from the National Institute on Aging rodent colony and young mice (3-months old) were obtained from Jackson Laboratories or Charles River Laboratories. Male mice were used in all experiments. A transgenic mouse model with the expression of high levels of human APP751 carrying V717I and K670M/N671L mutations (APP^Lon/Swe^) in neurons under control of a Thy1.2 promoter has been studied in different laboratories. The APP^Lon/Swe^ mice developed amyloid plaques associated with an overproduction of Aβ42 in the neocortex and working memory deficits at the age of 3 months, and the plaque formation spread to the hippocampus and thalamus region at 5–7 months^48,94^. This study used 3–6-month-old APP^Lon/Swe^ as well as age-matched non-transgenic mice. All mice were kept in a temperature- and humidity-controlled environment with a 12 h:12 h light:dark cycle and were provided ad libitum access to food and water.

### Lentivirus preparation

The protocol for the preparation of lentiviruses was previously described^88^. The FUW lentiviral vector expressing BAMN factors and EGFP is under the control of TetO promoter and M2rtTA under the control of ubiquitin promoter. HEK293T cells were plated in a poly-l-ornithine-coated 10-cm dish at a density of 6 × 10^6^ and co-transfected with 5 µg lentiviral transfer vector, 4 µg packaging plasmid (psPAX2) and 2.5 µg envelope plasmid (pMD2.G) the next day using Lipofectamine 2000 in OptiMEM medium (Thermo Fisher Scientific). After a 6-h incubation of the Lipofectamine 2000–DNA mixture in OptiMEM, the transfection medium was replaced with fresh DMEM supplemented with GlutaMAX, 2% FBS, 1% penicillin–streptomycin, 1% HEPES and 0.1% β-mercaptoethanol. The cell supernatants containing lentiviral particles were harvested after 24 h and stored at 4 °C. The cells were replenished with fresh DMEM medium plus 2% FBS and cultured for an additional 24 h. The supernatants were then harvested and pooled with the first collection. To remove cell debris, the supernatants were centrifuged at 400*g* for 5 min and passed through 0.45-µm syringe filters. The clear virus-containing media can be stored at 4 °C for about one week. Alternatively, for long-term storage, the virus-containing media were spun by ultracentrifugation for 90 min at 25,000 rpm and 4 °C to pellet the viruses. The viruses were resuspended in DMEM medium plus 2% FBS, and small aliquots were snap-frozen and stored at −80 °C.

### Direct generation of neurons from human fibroblasts

Human adult fibroblasts were plated at a density of 200 × 10^3^ cells per well of a six-well plate coated with poly-l-ornithine. The next day (Day 0), the fibroblasts were infected with lentiviruses expressing BAMN factors and M2rtTA with or without EGFP by incubation for 24h with the diluted virus-containing medium in DMEM supplemented with GlutaMAX, 2% FBS, 1% penicillin–streptomycin, 1% HEPES and 0.1% β-mercaptoethanol plus 4 µg ml^−1^ polybrene. On Day 1 the virus-containing medium was discarded and replaced with fresh fibroblast culture medium plus 1 µg ml^−1^ doxycycline. Puromycin (0.5 μg ml^−1^) was added on Day 2 for selection for 48 h. On Day 4 the transduced cells were subjected to PSA-NCAM+ selection following the manufacturer’s instructions. Briefly, 0.05% trypsin–EDTA was added to the cells for 5 min at 37 °C, to dissociate them from the surface, neutralized with fibroblast culture medium, followed by centrifugation at 300*g* for 5 min at room temperature to pellet the cells. The pelleted cells were resuspended in autoMACS buffer and labelled with anti-PSA-NCAM-APC (Miltenyi Biotec) for 10 min at 4 °C in the dark. Following a wash with autoMACS buffer and centrifugation at 300*g* for 10 min, the cells were incubated with anti-APC MicroBeads (Miltenyi Biotec) for 15 min at 4 °C in the dark. Unbound beads were then washed off and the cells were resuspended in autoMACS buffer for subsequent flow cytometry analysis and separation of magnetically PSA-NCAM-labelled and unlabelled cells. The PSA-NCAM+ cells were re-plated at a density of 50 × 10^3^ cells cm^−2^ on a plate coated with 5 μg ml^−1^ vitronectin (VTN-N; Thermo Fisher Scientific) and 1 μg ml^−1^ laminin (rhLaminin-521; Corning). The cells were cultured in fibroblast culture medium plus 1 µg ml^−1^ doxycycline and the next day switched to reprogramming medium (DMEM/F12:neurobasal (1:1) medium plus 2% B-27, 1% N-2, 0.25% GlutaMAX and 1% penicillin–streptomycin) supplemented with small molecules: 1 µg ml^−1^ doxycycline, 5 µM forskolin, 10 µM SB 431542, 2 µM dorsomorphin and 2 µM XAV939. After one week, 10 ng ml^−1^ BDNF and NT-3 were added to the reprogramming medium. Half of the medium was replaced with fresh medium every two or three days. After eight days, the cells were switched to maturation medium (BrainPhys neuronal medium plus 2% B-27, 1% N-2, 0.25% GlutaMAX and 1% penicillin–streptomycin) supplemented with 1 µg ml^−1^ doxycycline, 5 µM forskolin, 10 µM SB 431542, 2 µM dorsomorphin and 10 ng ml^−1^ BDNF and NT-3, and cultured for an additional 15–22 days. Half of the medium was replaced with fresh medium every three or four days. The efficiency of transdifferentiation of human fibroblasts into tNeurons was measured as the percentage of remaining transduced cells that express Tuj1, NeuN and MAP2 and the percentage of EGFP^+^ cells showing neuron-like morphology.

### TMT quantitative proteomics

Flash-frozen cell pellets were lysed in 8 M urea buffer (8 M urea, 150 mM NaCl, 50 mM HEPES pH 7.5, 1×EDTA-free protease inhibitor cocktail (Roche) and 1×PhosSTOP phosphatase inhibitor cocktail (Roche)). The lysates were clarified by centrifugation at 17,000*g* and 4 °C for 15 min. The protein concentration of the supernatant was determined using the bicinchoninic acid assay (Thermo Fisher Scientific). To reduce and alkylate cysteines, 100 µg protein was sequentially incubated with 5 mM Tris(2-carboxyethyl)phosphine hydrochloride for 30 min, 14 mM iodoacetamide for 30 min and 10 mM dithiothreitol for 15 min. All reactions were performed at room temperature. Next, proteins were chloroform–methanol precipitated and the pellet was resuspended in 200 mM 4-(2-hydroxyethyl)-1-piperazinepropanesulfonic acid (EPPS) pH 8.5 buffer. LysC (Wako) was added to the samples (1:100; LysC:protein), which were then incubated overnight at room temperature in an orbital shaker at 1,500 rpm. The following day the samples were trypsin digested (1:75 trypsin:protein) for 5 h at 37 °C in an orbital shaker at 1,500 rpm. After digestion, the samples were clarified by centrifugation at 17,000*g* for 10 min. A quantitative colorimetric peptide assay (Thermo Fisher Scientific) was used to determine the peptide concentration in the supernatant. For tandem mass tag (TMT) labelling, peptides from tNeuron samples were labelled with TMTpro-16plex tags according to a previously described protocol^95–97^. Briefly, 25 μg of peptides were diluted to 1 μg μl^−1^ in 200 mM EPPS pH 8.5; acetonitrile was added to the samples (final concentration of 30%), followed by the addition of 50 μg of each TMT reagent. After incubation at room temperature for 1 h, the reaction was stopped by the addition of 0.3% hydroxylamine (Sigma) for 15 min at room temperature. Extra information regarding both TMT sample labels is included in Supplementary Table 4. After labelling, all samples were combined, desalted with tC18 SepPak solid-phase extraction cartridges (Waters) and dried in a SpeedVac vacuum concentrator. Next, the desalted peptides were resuspended in 5% acetonitrile and 10 mM NH_4_HCO_3_ pH 8 and fractionated via basic pH reversed-phase chromatography using a high-performance liquid chromatography system equipped with a 3.5 µm Zorbax 300 Extended-C18 column (Agilent). Fractions were collected in a 96-well plate and combined into 24 samples. Twelve of these were desalted using a C18 Stop and Go Extraction Tip (STAGE-Tip)^98^ and dried in a SpeedVac. Finally, the peptides were resuspended in 1% formic acid and 3% acetonitrile, and analysed by liquid chromatography mass spectrometry, using the LC-MS^3^ method, in an Orbitrap Fusion Lumos (Thermo Fisher Scientific) system equipped with field asymmetric ion mobility spectrometry interface and running in RTS-MS3 mode^99–101^. More information regarding all mass spectrometry parameters is included in Supplementary Table 4. A suite of in-house pipelines (GFY‐Core version 3.8, Harvard University) was used to obtain final protein quantifications from all RAW files collected. RAW data were converted to mzXML format using a modified version of RawFileReader (5.0.7) and searched, using the search engine Sequest or Comet^102–104^, against a human target-decoy protein database (UP000005640, release-2019_04, downloaded from UniProt in June 2019) that included the most common contaminants. The precursor ion tolerance was set at 20 ppm and product ion tolerance at 1 Da. Cysteine carbamidomethylation (+57.0215 Da) and TMT tag (+304.2071 Da for TMTpro-16pex) on lysine residues and peptide amino termini were set as static modifications. Up to two variable methionine oxidations (+15.9949 Da) and two miscleavages were allowed in the searches. Peptide-spectrum matches were adjusted to a 1% false-discovery rate with a linear discriminant analysis^105^ and proteins were further collapsed to a final protein-level false-discovery rate of 1%. TMT quantitative values we obtained from MS3 scans. Only those with a signal-to-noise ratio of >100 and an isolation specificity of >0.7 were used for quantification. Each TMT was normalized to the total signal in each column. Quantifications included in Supplementary Table 4 are represented as relative abundances. RAW files will be made available on request. The data have been deposited in the ProteomeXchange Consortium via the PRIDE^106^ partner repository with the dataset identifier PXD059089. TMT-proteomics revealed a total of 6,015 proteins with more than two unique peptides, allowing us to identify the top proteomic hits affected by ageing and sAD. Biological pathway and gene ontology enrichment analysis were performed using ClueGo (Cytoscape plug-in), Enrichr or STRING.

### Caspase-3/7 activation

To detect apoptosis in tNeurons, we incubated cells with the caspase-3/7 substrate FAM-DEVD-FMK (ImmunoChemistry), one of the fluorochrome-labelled inhibitors of caspases that covalently and irreversibly binds to the active caspases. The green fluorescence intensity is a direct measurement of caspase-3/7 activity. Human tNeurons were treated with DMSO or 0.5 mM LLOME for 1 h to measure the lysosome-mediated apoptosis or pretreated with 3.1 µM C381, followed by LLOME treatment to assess the rescue effect of C381 on lysosome-mediated apoptosis. The FAM-DEVD-FMK reagent was reconstituted in DMSO and stored at −20 °C. When cells were available, the FAM-DEVD-FMK reagent was diluted with PBS (1:5 ratio) and added to cell culture medium at a dilution of 1:30 to form 1×staining solution. The cells were incubated with the FAM-DEVD-FMK solution for 30 min at 37 °C. After a rinse with Apoptosis Buffer, the cells were fixed with 4% paraformaldehyde (PFA) for 15 min and counterstained with 5 μg ml^−1^ Hoechst dye for confocal microscope imaging.

### Fluorescence-conjugated dextran assay to measure lysosomal acidification

Fluorescein isothiocyanate (FITC)-conjugated dextran at 40 kDa (Thermo Fisher Scientific) was reconstituted in water and stored at −20 °C. Human tNeurons were seeded at a concentration of 5 × 10^4^ cells per well of a 24-well plate or 5 × 10^3^ cells per well of a 96-well plate. The cells were incubated with 0.5 mg ml^−1^ FITC–dextran for 4 h at 37 °C and rinsed with PBS, followed by a 20-h chase in fresh culture medium to accumulate dextran in late endosomes and lysosomes. This was followed by treatment with DMSO or 0.25 mM LLOME for 30 min. The cells were fixed with 4% PFA for 15 min and prepared for imaging with a confocal microscope and CLARIOstar plate reader.

### Magic Red cathepsin-B assay to measure lysosomal proteolysis

Magic Red cathepsin-B substrate, MR-(RR)2, containing arginine-arginine (RR) sequence was reconstituted in DMSO and stored at −20 °C. When cells were available for an experiment, MR-(RR)2 was diluted with water (1:10 ratio) and added to cell culture medium at a dilution of 1:25 to form 1×staining solution. Active cathepsin-B cleaves MR-(RR)2 and emits fluorescence with an optimal excitation of 592 nm and emission of 628 nm. To test lysosomal proteolytic capacity, human tNeurons were incubated with MR-(RR)2 staining solution for 30 min at 37 °C, followed by DMSO or 0.25 mM LLOME treatment for 30 min. To evaluate the pharmacological rescuing effect, cells were pretreated with 3.1 µM C381 for seven days before the cells were loaded with MR-(RR)2. The cells were treated with 0.25 mM LLOME for 30 min and then fixed with 4% PFA for 15 min for imaging with a confocal microscope and CLARIOstar plate reader.

### Mitochondrial membrane potential

To detect mitochondrial membrane potential in human tNeurons, we used TMRE reagent (Abcam), which accumulates in functional and polarized mitochondria according to Δψm. A stock solution of 1 mM TMRE reagent was prepared by reconstituting TMRE reagent in DMSO and stored at −20 °C. After culturing tNeurons for five weeks, the cells were pretreated with DMSO, 50 μM FCCP or 0.25 mM LLOME for 10 min. Then TMRE reagent was added to fresh cell culture medium at a dilution of 1:1,000 along with FCCP or LLOME. To incubate the cells with TMRE at a final concentration of 500 nM for 30 min at 37 °C, half of the old culture medium was replaced with the TMRE-containing medium. The cells were then rinsed twice with pre-warmed 0.2% BSA in PBS and positioned in the CLARIOstar plate reader for fluorescence measurements at the optimal acquisition parameters (excitation of 549 nm and emission of 575 nm).

### ELISA

To measure Aβ42 levels, human tNeurons were trypsinized, washed with ice-cold PBS and pelleted by centrifugation at 1,000*g* for 5 min. The cells were then lysed with RIPA buffer (25 mM Tris–HCl pH 7.5, 150 mM NaCl, 1% NP-40, 0.5% sodium deoxycholate) supplemented with protease inhibitors (Roche). The protein concentrations were determined using the bicinchoninic acid assay (Thermo Fisher Scientific). For the Aβ42 assay, we used a human Aβ42 ELISA kit (Thermo Fisher Scientific) to detect and quantify the levels in total tNeuron lysates. Briefly, 50 µl of the cell lysates were added to each well of a 96-well plate, followed by incubation with anti-Aβ42 for 3 h, anti-rabbit IgG horseradish peroxidase for 30 min and stabilized chromogen for 30 min. The plate was analysed according to the manufacturer’s protocol and the Aβ42 values were normalized to the total protein concentration of lysates. Two independent experiments and cells from two HCs and patients with AD with three technical replicates (wells) were performed in this experiment.

### Calcium imaging

Cal-520–dextran conjugate at 3 kDa (AAT Bioquest) is a calcium-sensitive dye for detecting intracellular calcium levels, particularly in the compartmentalized organelles. Cal-520 was reconstituted in DMSO, aliquoted into single-use volumes and stored at −20 °C. Cells were incubated on sterile multi-chamber glass-bottomed slides for five weeks and then incubated with 5 μM Cal-520 and 0.1 μM LysoTracker Red DND-99 (Thermo Fisher Scientific) for 2 h at 37 °C. After washing off excess dye using PBS, the cells were prepared for live-cell imaging in a 37 °C incubation chamber with 5% CO_2_ on a Zeiss LSM 980 microscope. For Cal-520, an excitation wavelength of 490 nm and emission wavelength of 525 nm were used; for LysoTracker Red DND-99, excitation of 577 nm and emission of 590 nm were used.

### Mice brain perfusion and tissue processing

Mice were anaesthetized with 2.5% (vol/vol) avertin (Sigma-Aldrich). Transcardial perfusion with 50 ml cold PBS was performed using a peristaltic pump with the perfusate flow rate not exceeding 10 ml min^−1^. Brain tissue processing was performed as described previously^50,107^. Hemibrains were isolated and fixed overnight in 4% PFA at 4 °C before transferring to 30% sucrose in PBS at 4 °C for preservation. The hemibrains were cryosectioned coronally on a freezing-sliding microtome at a thickness of 40 μm and the free-floating sections were stored in cryoprotectant (40% PBS, 30% glycerol and 30% ethylene glycol) and kept at −20 °C until staining.

### Immunofluorescence and image acquisition and analysis

Human adult fibroblasts were fixed with 4% PFA for 15 min at room temperature, permeabilized with 0.3% Triton X-100 in PBS for 5 min or cold methanol on ice for 20 min and blocked with pre-warmed 5% BSA in PBS for 45 min at room temperature with shaking. The fibroblasts were incubated overnight in a moisture chamber at 4 °C with primary antibodies in 5% BSA. The following antibodies were used: anti-CHMP2B (1:400; Proteintech, 12527-1-AP), anti-galectin-3 (1:1,000; BioLegend, 125401), anti-γ-H2AX (1:200; Millipore Sigma, 05-636), anti-H3K9me3 (1:500; Abcam, ab8898), anti-H4K16ac (1:300; Thermo Fisher Scientific, MA5-27794), anti-LAMP1 (1:2,000; Cell Signaling Technologies, 9091), anti-LAMP2 (1:200; DSHB, H4B4), anti-p62/SQSTM1 (1:400; Abcam, ab56416), anti-S100A4 (1:200; Abcam, ab124805), anti-ubiquitin (1:200; LifeSensors, AB120) and anti-vimentin (1:400; Cell Signaling Technologies, 5741). The fibroblasts were washed three times with PBS and incubated with fluorophore-conjugated secondary antibody solution (anti-mouse, anti-rabbit and anti-rat; 1:500, Thermo Fisher Scientific) in the dark for 1 h at room temperature. Finally, the fibroblasts were washed three times with PBS, mounted with DAPI containing ProLong glass antifade mountant (Thermo Fisher Scientific) and air-dried overnight before imaging.

Human tNeurons were subjected to two-step fixation as follows: (1) half of the culture medium was removed and replaced with an equivalent volume of 4% PFA, followed by incubation at room temperature for 5 min and (2) the old solution was removed and replaced with 4% PFA, followed by incubation at room temperature for 15 min. The tNeurons were permeabilized with 0.3% Triton X-100 in PBS for 5 min at room temperature or cold methanol on ice for 20 min and blocked with pre-warmed 5% BSA in PBS at room temperature for 45 min. Next, the tNeurons were incubated overnight in a moisture chamber at 4 °C with the following primary antibodies in 5% BSA: anti-ASC/PYCARD (1:100; Santa Cruz, sc-514414), anti-GAP-43 (1:300; Novus Biologicals, NB300-143), anti-MAP2 (1:1,000; BioLegend, 822501), anti-NeuN (1:500; Abcam, ab177487), anti-NLRP3 (1:200; R&D systems, MAB7578), anti-p62/SQSTM1 (1:500; Proteintech, 18420-1-AP), anti-amyloid-β (1:100; Cell Signaling Technologies, 8243), anti-amyloid-β (1–42) (1:200; Enzo Life Sciences, ADI-905-804-100), anti-APP-CTF (1:200; BioLegend, 802803), anti-HGS (1:200; GeneTex, GTX101718), anti-Hsp27 (HspB1; 1:100; Proteintech, 18284-1-AP), anti-Hsp70 (1:1,000; Abcam, ab45133), anti-LC3B (1:100; Cell Signaling Technologies, 2775), anti-TDP-43 (1:1,000; Proteintech, 12892-1-AP), anti-pTau (AT8) (1:100; Thermo Fisher Scientific, MN1020), anti-pTau (S262) (1:200; FUJIFILM WAKO, 010-27123), anti-pTDP-43 Ser409/410 (1:300; Cosmo Bio, CAC-TIP-PTD-M01 and 1:200; BioLegend, 829901), anti-synapsin-1 (1:200; Abcam, ab64581), anti-tau (1:100; Aves Labs) and anti-β tubulin III (Tuj1) (1:500; Neuromics, CH23005 and 1:1,000; BioLegend, 801201). The tNeurons were washed three times with PBS and incubated at room temperature in the dark in fluorophore-conjugated secondary antibody solution for 1 h. The following secondary antibodies were used: anti-mouse, anti-rabbit, anti-rat and anti-chicken (1:500; Thermo Fisher Scientific). The samples were then washed three times with PBS, mounted with DAPI containing ProLong glass antifade mountant (Thermo Fisher Scientific) and air-dried overnight before imaging.

A detailed protocol for immunostaining of free-floating frozen and paraffin-embedded tissue sections is available^90^. Briefly, free-floating mouse tissue sections (30–50 µm thick) were collected and immunostained in multi-well plates. Mouse tissue sections were first rinsed three times with PBS and permeabilized with 0.3% Triton X-100 in PBS for 20 min at room temperature, followed by incubation with blocking buffer (10% normal donkey serum and 0.03% Triton X-100 in PBS) for 1 h at room temperature with shaking. Following PBS rinsing, the tissue sections were incubated overnight in a moisture chamber at 4 °C with the following primary antibodies in PBS plus 10% normal donkey serum: anti-amyloid-β (1–42) (1:100; Enzo Life Sciences, ADI-905-804-100), anti-CHMP2B (1:100; Proteintech, 12527-1-AP), anti-galectin-3 (1:50; R&D systems, AF1197), anti-Hsp70 (1:300; Abcam, ab45133), anti-LAMP1 (1:50; Santa Cruz, sc-19992) and anti-MAP2 (1:500; BioLegend, 822501). The samples were then incubated with fluorophore-conjugated secondary antibodies (anti-mouse, anti-rabbit, anti-goat or anti-chicken; 1:500; Thermo Fisher Scientific) for 1 h at room temperature. The tissue sections were washed twice with PBS, counterstained with Hoechst dye (1:2,000) and washed twice again with PBS before mounting with ProLong glass antifade mountant (Thermo Fisher Scientific). The samples were air-dried overnight before imaging.

Paraffin-embedded human brain tissues of the cerebral cortex were sectioned at a thickness of 10 μm. Deparaffinization was achieved by washing slides with xylenes twice (5 min each wash) and rehydrating via an ethanol (diluted in water) gradient (100, 95, 70 and 50%) wash (10 min each wash). Following heat-mediated antigen retrieval using Citrate buffer pH 6.0 (Sigma-Aldrich) for 30 min at 95 °C, the tissue sections were rinsed once with PBS, followed by incubation with blocking buffer (10% normal donkey serum and 0.03% Triton X-100 in PBS) for 2 h at room temperature. The slides were incubated overnight in a moisture chamber at 4 °C with the following antibody cocktails in blocking buffer: anti-amyloid-β (6E10) (1:100; BioLegend, 803001), anti-CHMP2B (1:100; GeneTex, GTX118181), anti-galectin-3 (1:50; R&D systems, AF1197 and 1:100; BioLegend, 125401), anti-Hsp70 (1:300; Abcam, ab45133), anti-LAMP2 (1:200; Abcam, ab213294) and anti-MAP2 (1:500; BioLegend, 822501). The tissue sections were then incubated with fluorophore-conjugated secondary antibodies (anti-mouse, anti-rabbit, anti-rat, anti-goat or anti-chicken; 1:500; Thermo Fisher Scientific) for 1 h at room temperature. After a PBS rinse, the slides were counterstained with Hoechst dye (1:2,000) and washed twice with PBS. Tissue sections were mounted with ProLong glass antifade mountant (Thermo Fisher Scientific) and allowed to complete air-dry overnight in the dark before imaging.

*Z*-series imaging (10–30 sections; 0.2–1 μm steps) acquisition was performed according to the experimental paradigm using a Zeiss LSM 700 or 980 confocal fluorescence microscope with ×20, ×63 and ×100 objectives. In each experiment, all groups were imaged using the same acquisition settings. The *z*-stack images were performed using maximum intensity projection to analyse the mean pixel intensity and determine a threshold to quantify the number of puncta in the cells using Fiji. For quantitative histology, between three and five separate sections were sampled using a ×20 objective and fluorescence signals were measured from entire image field to the mean fluorescence change. Tissue sections were imaged and analysed by blinded observers.

### TEM

Cells were cultured on Ibidi dishes (µ-Dish 35 mm, high Grid-50 Glass Bottom), followed by fixation in Karnovsky’s fixative—2% glutaraldehyde (EMS, catalogue number 16000) and 4% PFA (EMS, catalogue number 15700) in 0.1 M sodium cacodylate (EMS, catalogue number 12300) pH 7.4—for 1 h, chilled and sent to Stanford’s CSIF on ice. They were then post fixed in cold 1% osmium tetroxide (EMS, catalogue number 19100) in water and allowed to warm for 2 h in a hood, washed three times with ultra-filtered water, followed by en bloc staining in 1% uranyl acetate for 2 h at room temperature. The samples were then dehydrated in a series of ethanol washes (10 min each) at room temperature (30, 50, 70 and 95% ethanol, followed by two washes with 100% ethanol) and finally a 10-min wash with propylene oxide. The samples were infiltrated with EMbed-812 resin (EMS, catalogue number 14120), mixed 1:1 and 2:1 (2 h each) with propylene oxide. The samples were then placed into EMbed-812 for 2 h, after which they were moved to flat moulds with labels and fresh resin, and baked overnight in a 65 °C oven. Cells of interest were located using the grid pattern, cut out with a gem saw, remounted on pre-labelled resin blocks with fresh resin and allowed to polymerize overnight again. Once fully polymerized, the glass coverslip was etched away using hydrofluoric acid for 20 min. Using the finder, grid patterns left behind the block faces were trimmed down allowing for serial sectioning of the cells of interest. Sections of about 90 nm were cut, picked up on formvar–carbon-coated slot Cu grids and stained for 40 s in 3.5% uranyl acetate in 50% acetone, followed by staining with 0.2% lead citrate for 6 min. A JEOL JEM-1400 120 kV microscope was used to view the samples and photos were taken using a Gatan Orius 2 k × 2 k digital camera.

### Cytokine profiling analysis on neuronal conditioned medium using Luminex multiplex analysis

Cytokine profiling of the conditioned medium was used to analyse the secretion of inflammatory factors by tNeurons of healthy donors and patients with AD as previously described^91^. Conditioned medium was collected 48 h after the last medium change (at PID 38) of 1 ml per well of neuronal maturation medium in a 12-well plate, and centrifuged at 10,000*g* and room temperature for 10 min to pellet out particulates. For the human 80 plex panel (EMD-Millipore), a minimum of 200 µl supernatant was stored at −80 °C. Cell-free medium was also collected to monitor the background fluorescence. Cell numbers were determined by an automated cell counter for normalization of the cytokine levels. The setup of cytokine profiling assay was performed according to the manufacturer’s instructions. Briefly, the samples were mixed with antibody-linked magnetic beads on a 96-well plate and incubated overnight at 4 °C with shaking. Cold and room temperature incubation steps were performed on an orbital shaker at 500–600 rpm. The plates were washed twice with wash buffer in a Biotek ELx405 washer. Following incubation with biotinylated detection antibody for 1 h at room temperature, streptavidin–PE was added to the samples, which were then incubated for 30 min with shaking. The plates were washed as above and PBS was added to the wells for reading in a Luminex FlexMap3D instrument with a lower bound of 50 beads per sample per cytokine. Each sample was measured in duplicate. Custom Assay Chex control beads (Radix Biosolutions) were added to all wells. The analyses of all conditioned medium samples were performed using raw data (mean fluorescence intensity) rather than concentration (pg ml^−1^) to avoid calculating bias as recommended by the Stanford Human Immune Monitoring Center.

### CSF samples and protein discovery

We used the SOMAScan assay platform^108,109^ (SomaLogic Inc.) to measure the relative levels of 76 human proteins in CSF. This platform is based on modified single-stranded DNA aptamers (SOMAmer) capable of binding to specific protein targets with high sensitivity and specificity. We collected 79 CSF samples (50 HC and 29 AD samples) from a multi-ethnic cohort of older American adults (age range of 60–87 yr) between 2015 and 2020. The samples were stored at −80 °C and 150 µl aliquots of CSF were sent on dry ice to SomaLogic. The CSF samples were analysed via SOMAScan assay in five batches. To account for variation within and across batches, control, calibrator and buffer samples were added to each 96-well plate. Data normalization was conducted by the manufacturer following three stages. First, in hybridization control normalization, hybridization control probes were used to remove individual sample variance. Second, intraplate median signal normalization was performed, in which median normalization removed inter-sample differences within the plate. Finally, plate scaling and calibration removed variance across assay runs.

### Statistics and reproducibility

Quantification of fluorescence images was performed using the CLARIOstar plate reader software and open-source Fiji software. For each technical replicate, the fluorescence intensity of the background (cell-free solution or cell-free area in the image field) was subtracted from the intensity measurements. No statistical method was used to pre-determine the sample size. The representative images shown in Figs. 1d,g, 3g, 4b,c and Extended Data Figs. 2b, 3d, 6g,h, 9e,f were repeated at least two times. For quantifications of cytokine levels and human and mouse brain samples, the investigators were blinded to the group allocations. Statistical significance was determined using a two-sided Student’s *t*-test, one-way ANOVA or two-way ANOVA, based on the experimental design, using the GraphPad Prism Software. All values are expressed as box-and-whisker plots or the mean ± s.d. Differences between two groups were analysed using a two-sided Student’s *t*-test with Welch’s correction. Differences between multiple groups were analysed using a one-way or two-Way ANOVA, followed by Bonferroni’s post-hoc analysis. Differences were considered statistically significant for *P* < 0.05.

### Reporting summary

Further information on research design is available in the Nature Portfolio Reporting Summary linked to this article.

## Online content

Any methods, additional references, Nature Portfolio reporting summaries, source data, extended data, supplementary information, acknowledgements, peer review information; details of author contributions and competing interests; and statements of data and code availability are available at 10.1038/s41556-025-01623-y.

## Supplementary information

Supplementary Information Supplementary Figs. 1 and 2.

Reporting Summary

Supplementary Table 1 Supplementary Table 1. List of human adult fibroblasts, tNeurons and post-mortem brain tissues used for this study as well as sample information. Supplementary Table 2. Comparative analysis between this study and published AD genetics, transcriptomics and proteomics datasets. Supplementary Table 3. List of human CSF sample information. Supplementary Table 4. TMT quantitative proteomics analysis of human tNeurons from young, aged and AD donors. Supplementary Table 5. Antibodies used for this study. Supplementary Table 6. Cell lines and biological samples used for this study. Supplementary Table 7. Reagents used for this study. Supplementary Table 8. Plasmids used for this study. Supplementary Table 9. Software used for this study.

Supplementary Data 1 Statistical source data.

Supplementary Data 2 Statistical source data.

## Source data

Source Data Fig. 1 Statistical source data.

Source Data Fig. 3 Statistical source data.

Source Data Fig. 5 Statistical source data.

Source Data Fig. 6 Statistical source data.

Source Data Extended Data Fig. 1 Statistical source data.

Source Data Extended Data Fig. 2 Statistical source data.

Source Data Extended Data Fig. 3 Statistical source data.

Source Data Extended Data Fig. 5 Statistical source data.

Source Data Extended Data Fig. 6 Statistical source data.

Source Data Extended Data Fig. 7 Statistical source data.

Source Data Extended Data Fig. 8 Statistical source data.

Source Data Extended Data Fig. 9 Statistical source data.

Source Data Extended Data Fig. 10 Statistical source data.

## Data Availability

All proteomic source data of human tNeurons have been deposited and are publicly available at ProteomeXchange (accession number PXD059089). The mass spectrometry parameters, sample information, raw data and the comparison between our datasets and public genomic, transcriptomic and proteomic repositories are provided in Supplementary Tables 2 and 4. The cell lines, reagents, plasmids and software presented in the manuscript are reported in Supplementary Tables 5–9. The data, software, protocols, and key lab materials used and generated in this study are listed in a Key Resource Table alongside their persistent identifiers at *Zenodo*^110^. No code was generated for this study. The key resource table, supplementary datasets and source data related to this study are also available from *Zenodo*^110^. Source data are provided with this paper. All other data supporting the findings of this study are available from the corresponding author on reasonable request. Further requests for resources and reagents should be directed to the lead contact Judith Frydman (jfrydman@stanford.edu).
