# Supplementary material for: Proteostasis and lysosomal repair deficits in transdifferentiated neurons of Alzheimer’s disease
Source: Nat Cell Biol. 2025 Mar 26;27(4):619–32. doi: 10.1038/s41556-025-01623-y (PMC11991917; doi:10.1038/s41556-025-01623-y)
Supplement: Supplementary file 1 — Supplementary Figs. 1 and 2. [file 41556_2025_1623_MOESM1_ESM.pdf]

nature cell biology

Supplementary information https://doi.org/10.1038/s41556-025-01623-y

Proteostasis and lysosomal repair deficits in transdifferentiated neurons of Alzheimer's disease

In the format provided by the authors and unedited

Basal condition and chronic lysosomal damage in human tNeurons

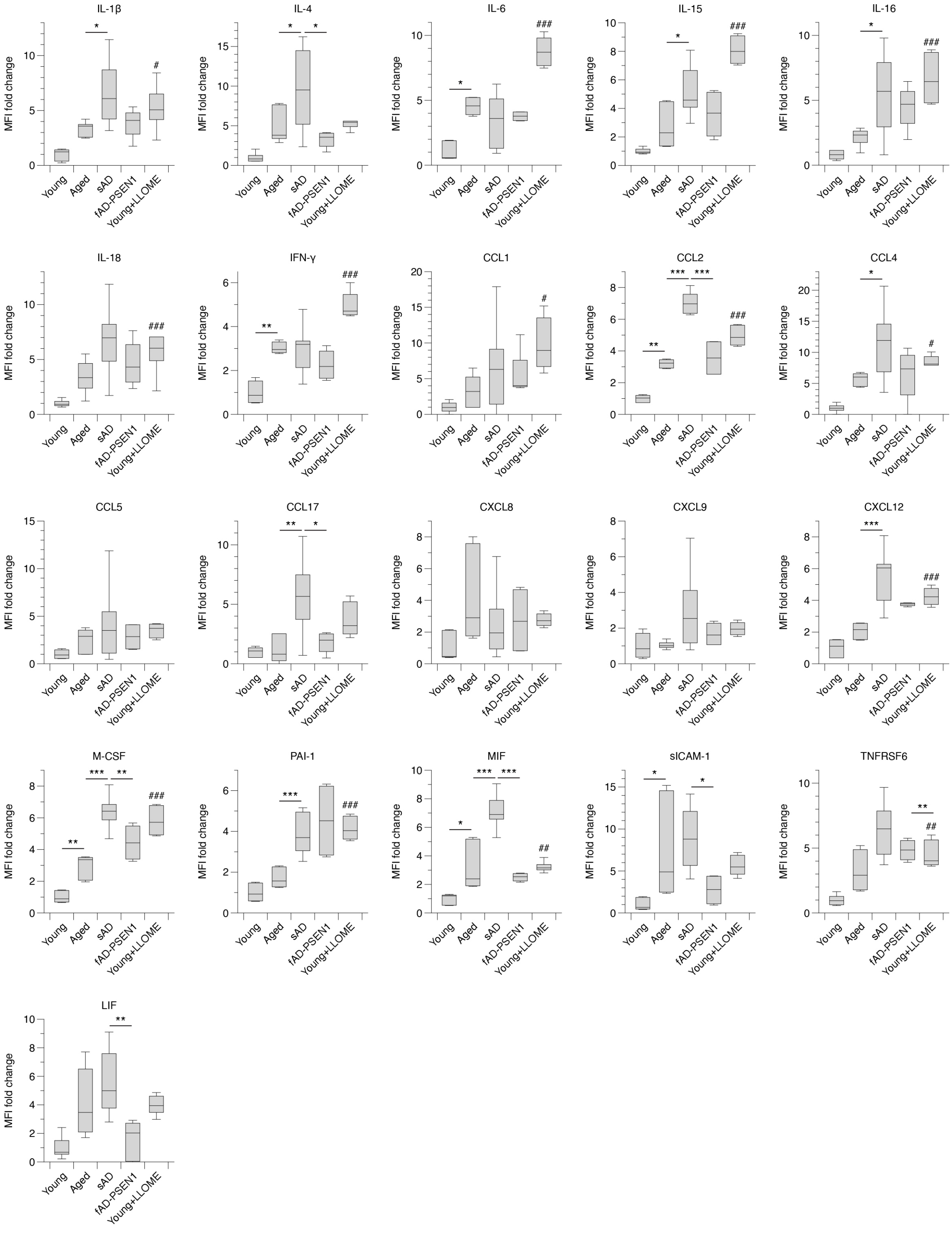

Supplementary Fig.1: Secretion of inflammatory factors from human tNeurons is linked to AD phenotypes.

Plots with statistical analysis of changes in inflammatory factor levels in the conditioned medium from young, aged, aged/sAD and fAD-PSEN1 tNeurons at basal conditions and young tNeurons with the treatment with 0.1 mM LLOME for 7 days, related to Fig. 5d,e.

Data show box-and-whisker plots of inflammatory profiling conducted in technical replicates and 2 to 6 cell lines in tNeurons. Box-and-whisker plots depict 5th, 25th, 50, 75th and 95th percentiles. Differences between groups are compared with One-Way ANOVA followed by Bonferroni post-hoc analysis. *P < 0.05, **P < 0.01 and ***P < 0.001. #P < 0.05, ##P < 0.01 and ###P < 0.001 as compared between young tNeurons with and without LLOME treatment.

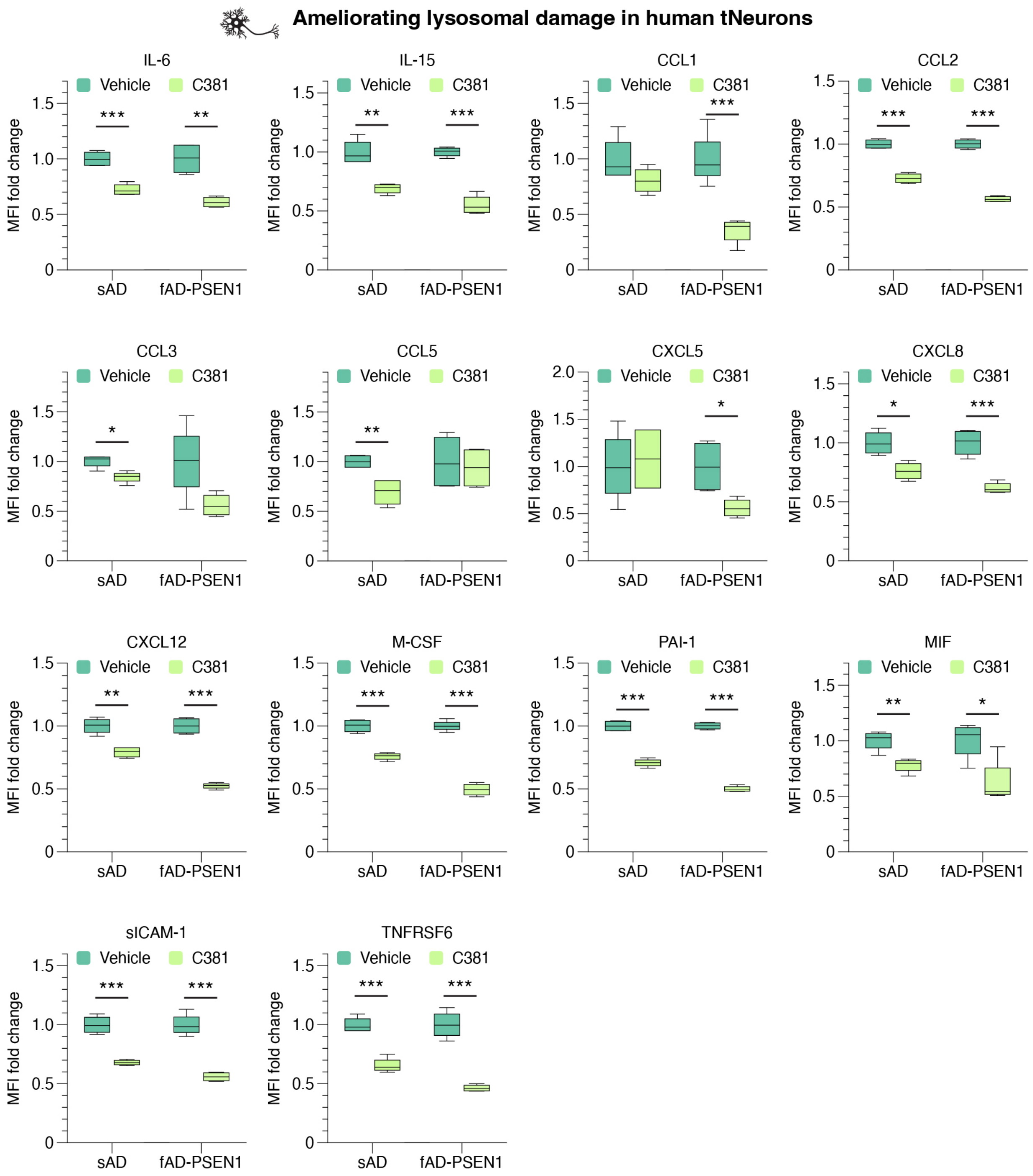

Supplementary Fig.2: Secretion of inflammatory factors from human tNeurons can be reduced by ameliorating lysosomal deficits.

Plots with statistical analysis of changes in inflammatory factor levels in the conditioned medium from aged/sAD and fAD-PSEN1 tNeurons with the treatment of DMSO (vehicle) or 3.1 µM C381 for 7 days as shown in Fig. 5g.

Data show box-and-whisker plots of inflammatory profiling conducted in technical replicates and 2 cell lines in tNeurons. Box-and-whisker plots depict 5th, 25th, 50, 75th and 95th percentiles. Differences between groups are compared with two-sided Student's *t*-test. $^*P < 0.05$, $^{**}P < 0.01$ and $^{***}P < 0.001$.
