# Supplementary material for: Proteostasis and lysosomal repair deficits in transdifferentiated neurons of Alzheimer’s disease
Source: Nat Cell Biol. 2025 Mar 26;27(4):619–32. doi: 10.1038/s41556-025-01623-y (PMC11991917; doi:10.1038/s41556-025-01623-y)
Supplement: Supplementary file 2 — Reporting Summary [file 41556_2025_1623_MOESM2_ESM.pdf]

nature portfolio

Corresponding author(s): Ching-Chieh Chou
Judith Frydman

Last updated by author(s): Nov 25, 2024

Reporting Summary

Nature Portfolio wishes to improve the reproducibility of the work that we publish. This form provides structure for consistency and transparency in reporting. For further information on Nature Portfolio policies, see our Editorial Policies and the Editorial Policy Checklist.

Statistics

For all statistical analyses, confirm that the following items are present in the figure legend, table legend, main text, or Methods section.

n/a	Confirmed	
☐	☒	The exact sample size (*n*) for each experimental group/condition, given as a discrete number and unit of measurement
☐	☒	A statement on whether measurements were taken from distinct samples or whether the same sample was measured repeatedly
☐	☒	The statistical test(s) used AND whether they are one- or two-sided<br>*Only common tests should be described solely by name; describe more complex techniques in the Methods section.*
☐	☒	A description of all covariates tested
☐	☒	A description of any assumptions or corrections, such as tests of normality and adjustment for multiple comparisons
☐	☒	A full description of the statistical parameters including central tendency (e.g. means) or other basic estimates (e.g. regression coefficient) AND variation (e.g. standard deviation) or associated estimates of uncertainty (e.g. confidence intervals)
☐	☒	For null hypothesis testing, the test statistic (e.g. *F*, *t*, *r*) with confidence intervals, effect sizes, degrees of freedom and *P* value noted<br>*Give P values as exact values whenever suitable.*
☒	☐	For Bayesian analysis, information on the choice of priors and Markov chain Monte Carlo settings
☒	☐	For hierarchical and complex designs, identification of the appropriate level for tests and full reporting of outcomes
☐	☒	Estimates of effect sizes (e.g. Cohen's *d*, Pearson's *r*), indicating how they were calculated

Our web collection on statistics for biologists contains articles on many of the points above.

Software and code

Policy information about availability of computer code

Data collection	Zeiss LSM 700 and 980 (confocal microscopy), CLARIOstar (plate reader), Luminex FLEXMAP 3D (cytokine), SOMAScan (CSF), JEOL JEM-1400 (TEM), Orbitrap Fusion Lumos Mass Spec (proteomics), FACSDiva software and Sony MA900 Multi-Application Cell Sorter
Data analysis	FIJI (v2.14.0), Prism 9, FlowJo 10.8.1, Cytoscape (v3.10.2) with ClueGo and STRING plug-in, GFY Core (v3.8), Enrichr, Morpheus

For manuscripts utilizing custom algorithms or software that are central to the research but not yet described in published literature, software must be made available to editors and reviewers. We strongly encourage code deposition in a community repository (e.g. GitHub). See the Nature Portfolio guidelines for submitting code & software for further information.

Data

Policy information about availability of data

All manuscripts must include a data availability statement. This statement should provide the following information, where applicable:

- Accession codes, unique identifiers, or web links for publicly available datasets
- A description of any restrictions on data availability
- For clinical datasets or third party data, please ensure that the statement adheres to our policy

All proteomic source data of human tNeurons have been deposited and are publicly available at ProteomeXchange (accession number: PXD040834). The mass spectrometry parameters, sample information, raw data and the comparison between our datasets and public genomic, transcriptomic and proteomic repositories are provided in Supplementary Tables.

Research involving human participants, their data, or biological material

Policy information about studies with human participants or human data. See also policy information about sex, gender (identity/presentation), and sexual orientation and race, ethnicity and racism.

Reporting on sex and gender	Biological samples were collected from healthy control donors and patients with Alzheimer's disease including biologically male and female.
Reporting on race, ethnicity, or other socially relevant groupings	N/A
Population characteristics	De-identified human fibroblasts, post-mortem prefrontal cortex and CSF samples were acquired from individuals of various ages and disease conditions. In the fibroblast samples, the age of healthy young donors ranged from 17 to 30 years old; the age of healthy aged donors ranged from 62 to 79 years old; the age of patients with sporadic AD ranged from 60 to 87 years old; the age of patients carrying PSEN1 mutations ranged from 31 to 56 years old. In the postmortem brain samples, the age of healthy control donors ranged from 41 to 80 years old; the age of patients with AD ranged from 65 to 93 years old. In the CSF samples, the age of healthy control donors ranged from 62 to 87 years old; the age of patients with AD ranged from 60 to 82 years old.
Recruitment	Cells and tissue samples were collected from repository resources. No human participant was specifically recruited for this study.
Ethics oversight	Cells and tissue samples were obtained under written consents from all subjects approved by Institutional Review Board of Stanford University.

Note that full information on the approval of the study protocol must also be provided in the manuscript.

Field-specific reporting

Please select the one below that is the best fit for your research. If you are not sure, read the appropriate sections before making your selection.

☒ Life sciences ☐ Behavioural & social sciences ☐ Ecological, evolutionary & environmental sciences

For a reference copy of the document with all sections, see nature.com/documents/nr-reporting-summary-flat.pdf

Life sciences study design

All studies must disclose on these points even when the disclosure is negative.

Sample size	No statistical analysis was used to pre-determine the sample size. Sample sizes were chosen to be similar or exceed those reported in the previous literatures in the field. Statistical analysis demonstrates that our sample sizes reveal significant differences between groups.
Data exclusions	No samples were excluded from data analyses.
Replication	Each experiment used cells or tissue samples at least from two individuals and at least repeated at least 2 times. The data were collected from multiple independent experiments every week or every other week, and showed successful reproducibility.
Randomization	This study retrospectively analyzed cells and tissue samples. Randomization was not applicable.
Blinding	Quantifications of cytokine levels and tissue sections were analyzed by blinded observers

Reporting for specific materials, systems and methods

We require information from authors about some types of materials, experimental systems and methods used in many studies. Here, indicate whether each material, system or method listed is relevant to your study. If you are not sure if a list item applies to your research, read the appropriate section before selecting a response.
